# Posterior enhancer (p-Enh) maintains early neuromesodermal progenitors bi-potency during gastrulation

**DOI:** 10.1186/s13619-025-00272-8

**Published:** 2025-11-15

**Authors:** Panpan Mi, Yingying Chen, Fengxiang Tan, Penglei Shen, Yun Yang, Mingzhu Wen, Yun Qian, Jichang Wang, Naihe Jing, Xianfa Yang

**Affiliations:** 1 Department of Histology and Embryology, Zhongshan School of Medicine, Sun Yat-Sen University, Guangzhou, 510080 China; 2 Key Laboratory for Stem Cells and Tissue Engineering (Sun Yat-Sen University), Ministry of Education, Guangzhou, 510080 China; 3 Guangzhou National Laboratory, Guangzhou International Bio Island, Guangzhou , Guangdong, 510005 China; 4 State Key Laboratory of Cell Biology, Center for Excellence in Molecular Cell Science, Shanghai Institute of Biochemistry and Cell Biology, Chinese Academy of Sciences, University of Chinese Academy of Sciences, Shanghai, 200031 China; 5 Guangzhou Medical University, Guangzhou, 511495 Guangdong China

**Keywords:** Gastrulation, Neuromesodermal progenitors, Enhancer, Cell fate determination, ST-Pheno

## Abstract

Vertebrate axis patterning requires precise control of the differentiation of neuromesodermal progenitors (NMPs), which generate spinal cord (SC) and presomitic mesoderm (PSM). Previously, we identified a gastrula-premarked posterior enhancer (p-Enh) that is essential for posterior tissue development by regulating somite and SC in organogenetic embryos, while its role in early NMPs cells remains elusive. Here, using a highly efficient in vitro differentiation system, we found that the genetic removal of p-Enh leads to the aberrantly up-regulated PSM-related genes during both PSM and SC differentiation. Time-resolved transcriptomic analysis and experimental characterization revealed the activated PSM transcriptomic signature arose from disorganized NMPs composition, with an over-representation of the T^high^SOX2^low^ NMPs subtype. Besides, through a newly developed bioinformatic tool, ST-Pheno, which effectively bridges the in vitro samples to in vivo embryonic phenotypes within spatiotemporal context*,* we determined that the over-produced T^high^SOX2^low^ NMPs subtype is predominantly enriched in the anterior primitive streak and adjacent mesoderm region at E7.5, which may disrupt the proper development of NMPs towards prospective PSM and SC, ultimately leading to the posterior development failure. In summary, this study demonstrates a critical role of p-Enh in regulating NMPs subtype composition, which will broaden the molecular understanding of mammalian embryogenesis.

**Supplementary Information:**

The online version contains supplementary material available at 10.1186/s13619-025-00272-8.

## Background

Gastrulation is a pivotal developmental event that generates the three germ layers—ectoderm, mesoderm, and endoderm—while establishing the embryonic body plan through coordinated lineage specification and cell fate decisions (Lawson et al. 1991; Tam and Behringer 1997; Bardot and Hadjantonakis 2020; Wang et al. 2023). Among the progenitor populations emerging during this stage, neuromesodermal progenitors (NMPs) have garnered particular interest due to their dual potential to generate both posterior spinal cord (SC) and presomitic mesoderm (PSM), thereby driving posterior body axis elongation (Tzouanacou et al. 2009; Shaker et al. 2021). NMPs emerge around embryonic day 7.5 (E7.5) in posterior regions such as the anterior primitive streak (APS) and the node-streak border, contributing to the continuous extension of the body axis in later organogenesis (Cambray and Wilson 2002, 2007; Henrique et al. 2015).

Gene regulatory networks (GRNs) orchestrate gene expression programs and establish cellular identity by coordinating signaling pathways and transcription factors during development (Davidson and Erwin 2006; Karlebach and Shamir 2008). In NMPs, the tightly controlled GRNs ensure balanced differentiation into neural and mesodermal lineages (Gouti et al. 2017; Koch et al. 2017). For instance, recent studies, particularly those employing single-cell transcriptomics, have identified key signaling pathways, including WNT, FGF, and retinoic acid (RA) and transcription factors, such as *T* (*Brachyury*), *Sox2*, *Cdx2*, and *Tbx6*, which act coordinately within these regulatory network (Nordström et al. 2006; Duester 2008; Niederreither and Dollé, 2008; Ribes et al. 2009; Wilson et al. 2009; Mazzoni et al. 2013; Cunningham et al. 2015; Koch et al. 2017; Gouti et al. 2017; Bolondi et al. 2024; Braccioli et al. 2025). Critically, mutual repression between T and SOX2 establishes a core circuit that maintains NMPs’ bi-potency (Henrique et al. 2015; Wymeersch et al. 2016; Koch et al. 2017). These regulatory networks stabilize NMPs within specific embryonic region, such as the caudal lateral epiblast and the chordoneural hinge, ensuring continuous and robust axial elongation (Cambray and Wilson 2002, 2007; Delfino-Machín et al. 2005; Kondoh and Takemoto 2012; Chen et al. 2024). Despite these advances, our understanding of how NMPs’ bi-potency is initiated, established, and stabilized at earlier developmental stages remains limited and warrants deeper investigation.

Epigenetic regulation shapes early embryonic development and lineage specification (Zhao et al. 2008; Li and Wang 2015). Recently, we and others have identified a gastrula-stage posterior enhancer (p-Enh) that exhibits chromatin accessibility and is pre-marked with H3K27ac in posterior epiblast cells at E7.5, the stage coinciding with NMPs emergence (Amin et al. 2016; Yang et al. 2019). Functional analyses have shown that p-Enh is critical for posterior tissue development, with its deletion reducing NMPs and PSM abundance at E9.5, acting both *in cis* to regulate *Cdx2* expression and *in trans* by interacting with SMAD4 protein (Chen et al. 2025; Amblard et al. 2025). However, the potential contribution of p-Enh to regulating early NMPs identity remains unclear.

To systematically dissect p-Enh’s functions in NMPs fate determination, we employed in vitro, in vivo*,* and in silico approaches and found that p-Enh knockout (p-Enh-KO) cells significantly altered NMPs transcriptional states, up-regulating the presence of a mesoderm-primed (T^high^SOX2^low^) NMPs sub-population. Consistently, time-resolved transcriptomics profiling confirmed this bias within NMPs and in their SC and PSM derivatives. Furthermore, ST-Pheno revealed that p-Enh loss induces the aberrant presence of this specific sub-population in the APS region of E7.5 gastrula. Experimental validation using p-Enh-KO embryos confirmed accumulation of T^high^SOX2^low^ cells in the APS and adjacent mesoderm, demonstrating that p-Enh deletion rewires the cellular composition of NMPs. Together, these findings identify p-Enh as an essential regulator that maintains proper NMPs formation and subsequent axial elongation.

## Results

### In vitro differentiation system recapitulates the developmental trajectory from pluripotent stem cell, NMPs, to prospective PSM and SC

**Fig. 1:**
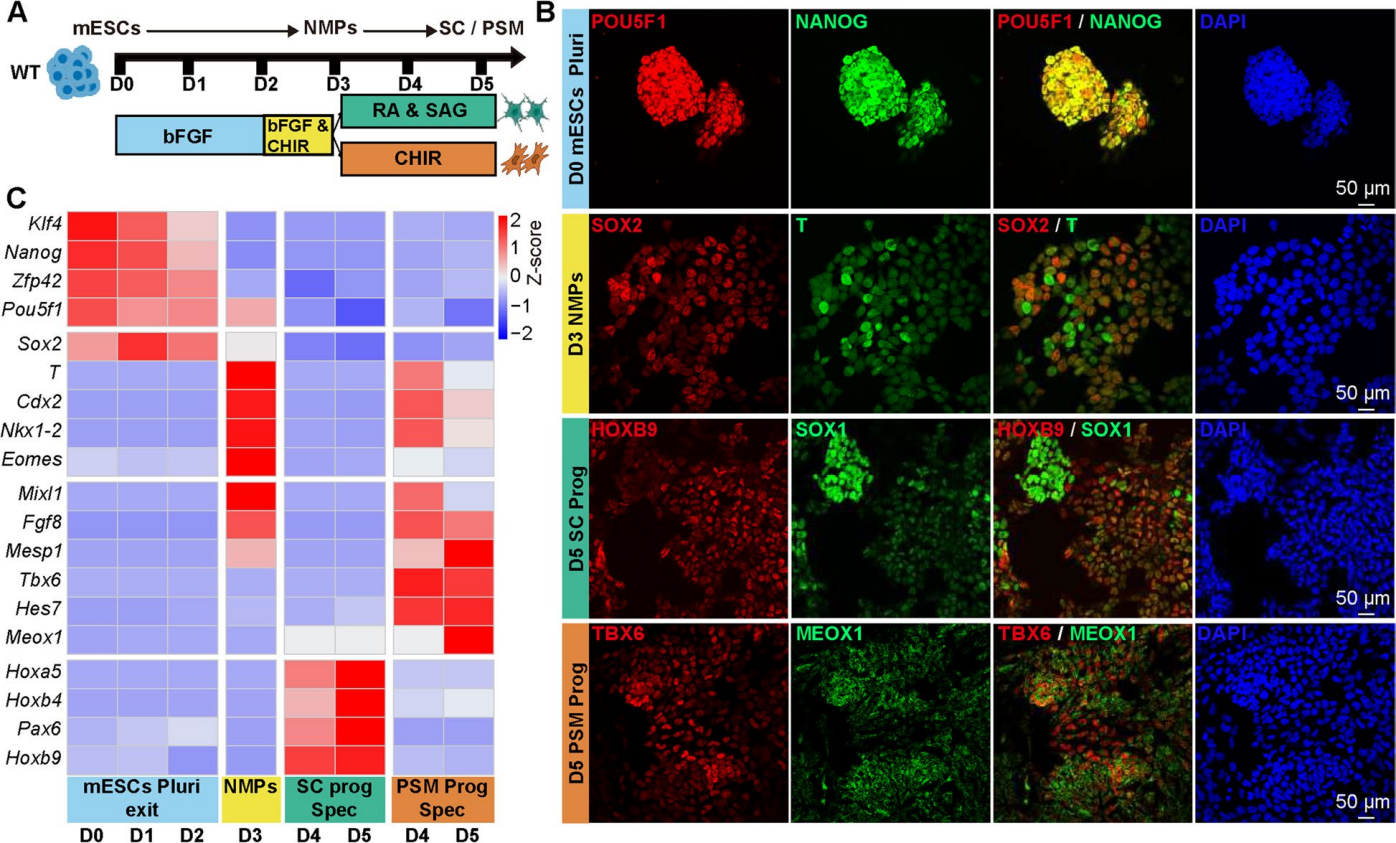
Differentiation of mESCs into SC and PSM via NMPs. **A** Schematic representation of the differentiation protocol used to generate SC and PSM progenitors from mESCs through a common NMPs intermediate. **B** Immunofluorescence analysis of pluripotency markers POU5F1 and NANOG at day 0 (D0); NMPs progenitor markers SOX2 and T at D3; SC progenitor markers HOXB9 and SOX1 at D5; and PSM markers TBX6 and MEOX1 at D5. Nuclei were counterstained with DAPI (blue). Scale bar, 50 μm. Pluri: Pluripotency; Prog: Progenitor. **C** Heatmap of normalized qPCR expression profiles based on Z-score (*n* = 3 biological replicates) showing expression dynamics associated with pluripotency, NMPs, SC, and PSM lineages over the course of differentiation (D0-D5). Spec: Specification

Robust access to developmentally relevant progenitor cells is crucial for mechanistic studies of vertebrate axial development (Kuzmicz-Kowalska and Kicheva 2020). In vivo, the scarcity of NMPs during early embryogenesis presents a significant barrier to their detailed molecular and functional characterization. To overcome this limitation and specifically enable the efficient, high-yield generation of NMPs and their differentiated derivatives, we employed a stepwise, temporally controlled in vitro differentiation system from established methodologies (Gouti et al. 2014) (Fig. 1A). As reported, this five-day-long differentiation can be divided into three stages: mESCs pluripotency exit (D0-D2), NMPs (D3), and SC/PSM progenitor specification (D4-D5) (Turner et al. 2014) (see Materials and methods). This in vitro platform thus provides a powerful and scalable model for dissecting the mechanisms governing NMPs biology and subsequent posterior axis formation.

This timed protocol faithfully models early developmental transitions with high temporal resolution and molecular fidelity. Immunofluorescence analysis revealed cellular transitions from highly organized pluripotent mESCs (POU5F1 and NANOG) (Yeo and Ng 2012) at D0 to bi-potential NMPs (co-expressing SOX2 and T) (Wymeersch et al. 2016) at D3, and ultimately to SC progenitors (HOXB9 and SOX1) (Wilmerding et al. 2021) or PSM progenitors (TBX6 and MEOX1) (Javali et al. 2017) by D5 (Fig. 1B). qPCR analysis of our time-resolved differentiation (Fig. 1C) confirmed dynamic marker expression: pluripotency markers (e.g., *Nanog*, *Klf4*) (Silva et al. 2009; Marzi et al. 2013) were gradually downregulated, NMPs-specific markers (e.g., *T*, *Nkx1-2*) (Edri et al. 2019) peaked at D3, mirroring in vivo transcriptome profiles, and representative SC (e.g., *Hoxb9*, *Hoxa5*) (Joksimovic et al. 2005) and PSM markers (e.g., *Tbx6*, *Hes7*) (Hayashi et al. 2018; Zhang et al. 2022) were successfully activated by D5.

Furthermore, immunofluorescence enabled precise analysis at single-cell resolution, revealing the homogeneity of cellular states. Collectively, these results underscore our in vitro differentiation system as an exceptionally reliable and powerful model for studying cell fate determination and homeostasis of NMPs.

### p-Enh-KO activates mesoderm-related gene signatures during in vitro differentiation and boosts the enrichment of mesoderm-primed NMPs subtype

**Fig. 2:**
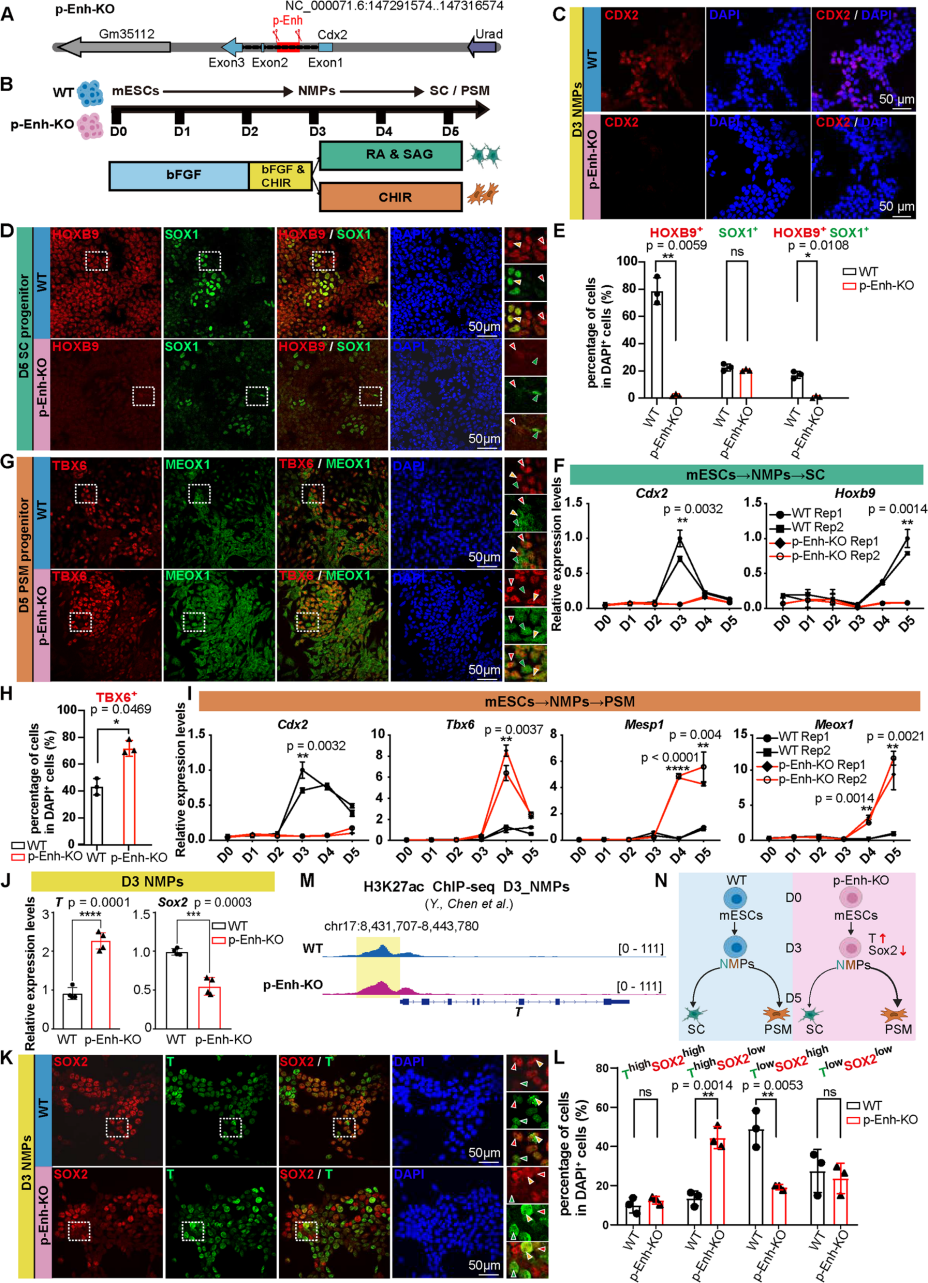
Deletion of p-Enh alters the intrinsic state of NMPs and disrupts downstream lineage specification. **A** Schematic of the *Cdx2* locus showing p-Enh enhancer (chr5: 147,304,002–147,305,268); the red scissor indicates CRISPR-mediated deletion of this enhancer in p-Enh-KO cells. **B** Differentiation protocol for WT and p-Enh-KO mESCs toward NMPs, SC and PSM fates. **C** Representative immunofluorescence of CDX2 (red) and DAPI (blue) in D3 NMPs from WT and p-Enh-KO groups. Scale bar, 50 μm. **D** Immunofluorescence of HOXB9 (red) and SOX1 (green) in D5 SC. Insets show magnified views of regions outlined by dashed white boxes. Red arrows indicate HOXB9 positive cells, green arrows show SOX1 positive cells, and yellow arrows mark double-positive cells. Scale bar, 50 μm. **E** Quantification of single-positive (HOXB9^+^ or SOX1^+^) and double-positive cells from (**D**), expressed as percentage of DAPI^+^ cells (*n* = 3). Statistical significance: ns, not significant; *p < 0.05; **p < 0.01; ***p < 0.001; ****p < 0.0001. This significance notation is used consistently throughout all figures. **F** Time course qPCR analysis of *Cdx2* and SC markers *Hoxb9* during D0–D5 differentiation (*n* = 2). **G** Immunofluorescence of TBX6 (red) and MEOX1 (green) in D5 PSM. Insets show magnified views of regions outlined by dashed white boxes. Red arrows indicate TBX6^+^ cells, green arrows show MEOX1^+^ cells, and yellow arrows mark double-positive cells. Scale bar, 50 μm. **H** Quantification of TBX6 positive cells in (**G**), shown as percentage of DAPI positive cells (*n* = 3). **I** Time course qPCR analysis of the PSM markers *Cdx2*, *Tbx6*, *Mesp1* and *Meox1* during D0-D5 differentiation (*n* = 2). **J** qPCR quantification of NMPs markers (*T* and *Sox2*) at D3 (*n* = 4). **K** Dual immunofluorescence staining of SOX2 (red) and T (green) in D3 NMPs. Insets show magnified views. Red arrows denote SOX2^high^ cells, green arrows indicate T^high^ cells, and yellow arrows mark double-positive cells expressing high levels of SOX2 and T. Scale bar, 50 μm. **L** Statistical quantification of T/SOX2 subpopulations (T^high^SOX2^low^, T^low^SOX2^high^, T^low^SOX2^low^, T^high^SOX2^high^) as percentage of total DAPI^+^ cells in (K) (*n* = 3). **M** IGV snapshots comparing H3K27ac ChIP-seq profiles at *T* locus between WT and p-Enh-KO in D3 NMPs. **N** Proposed Model: p-Enh deletion disrupts the intrinsic state of NMPs, reducing *Sox2* expression and elevating* T* levels. This imbalance impairs SC differentiation competence and biases cells toward PSM fates. Figure created with Biorender

To functionally characterize the role of p-Enh in NMPs cell fate determination, we first generated two independent p-Enh-KO mESCs lines with precise deletions of the enhancer region using the CRISPR/Cas9 system (Ran et al. 2013), preserving intact *Cdx2* coding sequences (Figs. 2A, S1A-B). Both clones maintained a normal pluripotent state, exhibiting uniform morphology and marker gene expression comparable to wild-type (WT) mESCs. Then, both WT and p-Enh-KO mESCs were subjected to in vitro differentiation, with comparative analysis of cellular states at distinct developmental stages (Fig. 2B). In consistent with previous reports (Chen et al. 2025; Amblard et al. 2025), the robust CDX2 expression in WT NMPs was largely abolished in D3 p-Enh-KO NMPs (Fig. 2C), confirming that the efficient removal of p-Enh. Next, to probe the potential effects of p-Enh knockout, we utilized Cellpose-based image segmentation (Stringer et al. 2020) to resolve cellular states and then quantify immunofluorescence results at single-cell resolution (Fig. S1C, see Materials and methods). As shown, quantitative immunofluorescence analyses at D5 revealed significantly reduced HOXB9-positive and HOXB9-SOX1 double-positive neural progenitors in p-Enh-KO SC group (Fig. 2D-E). qPCR analysis verified that the significant downregulation of caudal SC marker-*Hoxb9* in p-Enh-KO differentiated SC cells (Fig. 2F). Intriguingly, under mesoderm differentiation conditions, dual immunostaining for PSM marker genes, TBX6 and MEOX1 (Reijntjes et al. 2007; Javali et al. 2017), demonstrated an increased proportion of TBX6-positive PSM cells in the p-Enh-KO group (Fig. 2G-H). qPCR analysis further confirmed significantly upregulated expression of mesodermal markers (e.g., *Tbx6*, *Mesp1*) (Fig. 2I). Unlike the absence of PSM cells in p-Enh-KO organogenetic embryos (Chen et al. 2025), the aberrant upregulation of mesodermal markers in p-Enh-KO cells observed here indicates a previously unrecognized role for p-Enh element in directing stem cell differentiation, which may be masked by endogenous compensatory mechanisms in vivo but amplified by the forced differentiation cues in the in vitro differentiation system.

To track the differentiation origin of abnormal enrichment of PSM in p-Enh-KO group, we investigated the molecular features at an earlier stage (D3 NMPs), which are marked by the co-existence of SOX2 and T (Gouti et al. 2017; Koch et al. 2017). Surprisingly, qPCR analysis using bulk samples collected from D3 NMPs revealed a severe disruption of NMPs signatures in D3 p-Enh-KO NMPs, manifesting as elevated *T* (p = 0.0001) but reduced *Sox2* expression (p = 0.0003) compared to WT control (Fig. 2J). Detailed exploration for cell composition in NMPs population through double staining of SOX2 and T showed a marked shift in p-Enh-KO cell populations: with an increased proportion of T^high^SOX2^low^ cells and a significantly decreased proportion of T^low^SOX2^high^ cells relative to the WT control group (Fig. 2K-L). To further investigate the underlying epigenetic mechanisms that may contribute to these cellular changes, we performed a comprehensive, genome-wide analysis of H3K27ac ChIP-seq data from D3 WT and p-Enh-KO samples (Chen et al. 2025). This analysis revealed a widespread increase in promoter-associated H3K27ac in the p-Enh-KO group, with 1940 genomic regions showing enhanced acetylation compared to WT, including key genes involved in mesoderm and NMPs development (Fig. S1D-J). For example, the increased H3K27ac deposition specifically at the *T* promoter and the nearby upstream genomic region in p-Enh-KO NMPs compared with WT (Fig. 2M). Given the pivotal roles of T in regulating PSM differentiation (Koch et al. 2017), the boosted presence of T^high^SOX2^low^ cells in p-Enh-KO group could enhance the differentiation bias towards PSM fate, which leads to the upregulation of PSM markers in D5.

To further investigate the molecular basis underlying the mesodermal fate bias observed in p-Enh-KO NMPs, we performed NMPs differentiation using Cdx2-knockout (Cdx2-KO) mESCs (Chen et al. 2025). qPCR analysis of *Cdx2* expression revealed that both Cdx2-KO and p-Enh-KO mESCs exhibited significantly reduced levels relative to WT during differentiation (Fig. S1K and S1L). At the D3 NMPs stage, the expression levels of *Sox2* and *T* in Cdx2-KO cells showed no significant difference compared with WT controls (Fig. S1M), indicating that *Cdx2* deficiency does not recapitulate the mesoderm-biased phenotype seen in p-Enh-KO cells. This may be attributed to the *Cdx2*-independent mechanisms for p-Enh function (Chen et al. 2025) and also functional redundancy among Cdx family members (Savory et al. 2009), wherein *Cdx1* and *Cdx4* may compensate for the loss of *Cdx2*.

Collectively, these results demonstrate that p-Enh functions at an early NMPs stage by balancing NMPs subtype composition and maintaining the epigenetic landscape at key lineage-determining loci, and its loss leads to an increase in the mesoderm-primed sub-population (T^high^SOX2^low^) (Fig. 2N).

### p-Enh deletion leads to pre-activation of mesoderm related TF regulons at D3 NMPs

**Fig. 3:**
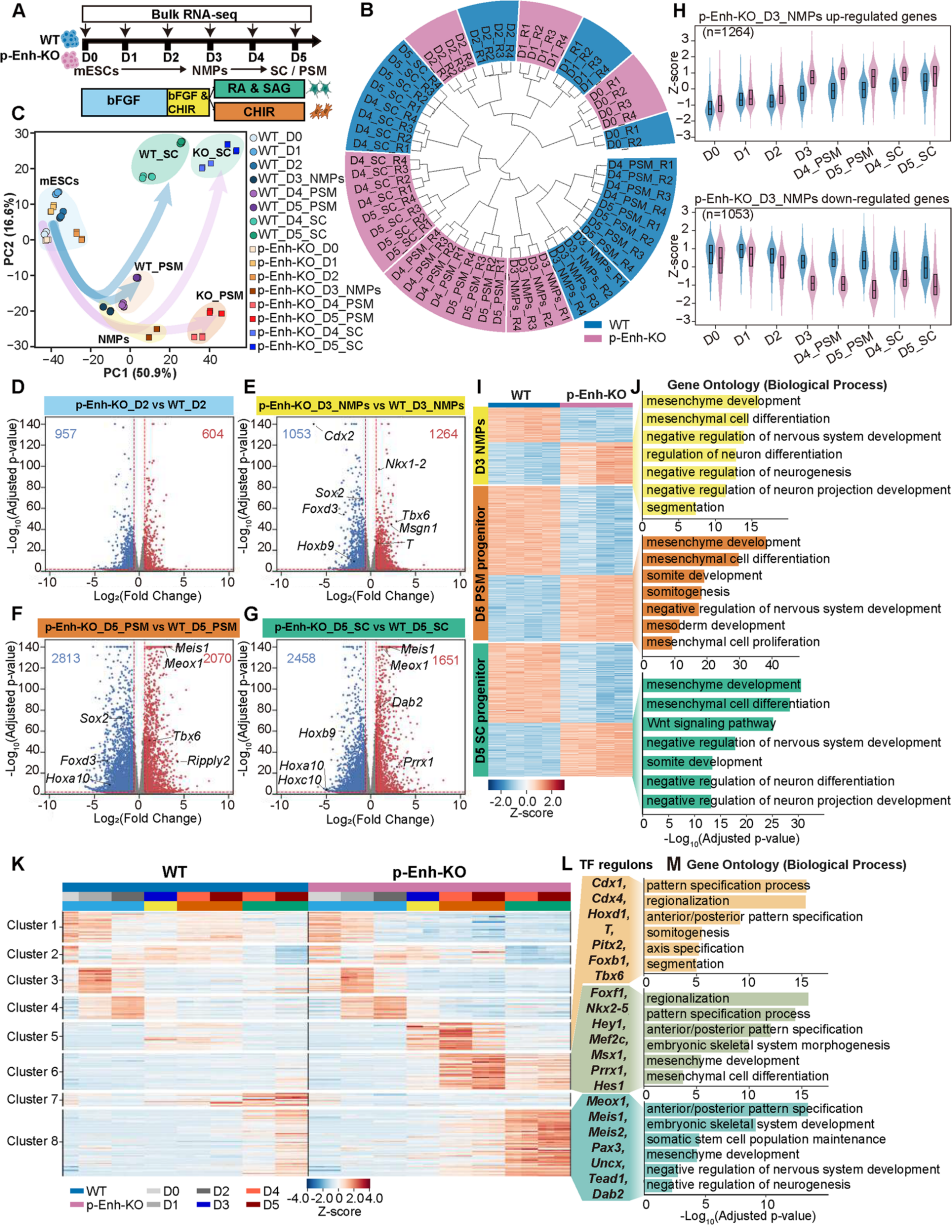
Time-resolved transcriptomics reveal p-Enh-KO diverts NMPs fate toward PSM with mesodermal regulons activation. **A** Schematic of bulk RNA-seq sampling during differentiation of WT and p-Enh-KO mESCs into NMPs, PSM, and SC lineages at daily intervals (D0-D5; *n* = 4). **B** Circular dendrogram of hierarchical clustering of all RNA-seq samples, with shaded colors denoting genotypes (blue, WT; pink, p-Enh-KO), biological replicates (R1-R4). **C** PCA scatter plot showing PC1 (50.9%) and PC2 (16.6%). Each spot represents an individual RNA-seq sample, with circles (WT) and squares (p-Enh-KO) grouped by sample stages (mESCs, NMPs, PSM, and SC). **D**-**G** Volcano plots of DEGs in p-Enh-KO vs. WT at D2(**D**), D3 NMPs (**E**), D5 PSM (**F**) and D5 SC(**G**). Red/blue dots denote genes significantly upregulated and downregulated in p-Enh-KO (| Log_2_(Fold Change) |> 1.5, adjusted *p*-value < 0.05); gene numbers and key regulators are labeled. **H** Violin plots showing Z-scored expression of 1,264 upregulated (upper) and 1,053 downregulated genes (bottom) in p-Enh-KO D3 NMPs across D0-D5 for WT (blue) and p-Enh-KO (pink). Boxplots indicate median and interquartile range. *p*-values are annotated. **I** Heatmaps of Z-scored DEGs in WT vs. p-Enh-KO cells at three developmental stages (D3 NMPs, D5 PSM and D5 SC). **J** GO enrichment analysis for genes upregulated in p-Enh-KO relative to WT at the stages shown in (**I**). Bars represent the -Log_10_(adjusted *p*-value) for each enriched term. **K** Heatmap of Z-scored regulon activity across differentiation (D0-D5) in WT vs. p-Enh-KO cells. Regulons were clustered into eight discrete modules (Clusters 1–8). **L** Examples of representative regulon TFs from Clusters 5, 6 and 8 in (**K**). **M** GO enrichment for Clusters 5, 6 and 8 in (**K**), shown as -Log_10_(adjusted *p-*value) for the enriched terms

To systematically evaluate the effects of p-Enh deletion, we performed time-course RNA-seq on both WT and p-Enh-KO cells across the differentiation toward SC and PSM lineages. (Fig. 3A). For each condition and time point, two high-quality biological replicates were prepared (Figs. 3A, S2). Correlation analysis with in vivo single-cell RNA-seq data showed that each in vitro stage closely matched its corresponding embryonic counterpart, highlighting the robustness and fidelity of our differentiation system (Fig. S3A) (Pijuan-Sala et al. 2019).Transcriptome-wide hierarchical clustering and principal component analysis (PCA) revealed a clear developmental trajectory in WT samples, progressing from embryonic stem cells to NMPs and ultimately to respective lineage derivatives (Fig. 3B-C). In contrast, p-Enh-KO samples formed distinct clusters separate from their WT counterparts, particularly from D3 onward (Fig. 3B-C). These data suggest that p-Enh deletion could disrupt the normal transcriptomic trajectory of NMPs differentiation.

Pairwise differentially expressed genes (DEGs) analysis revealed that the transcriptional distinctions between p-Enh-KO and WT control progressively increased, as reflected by a growing number of DEGs (Fig. 3D-G). To specify, at D2, 604 up-regulated genes and 957 down-regulated genes can be detected in p-Enh-KO group (Fig. 3D). By D3, p-Enh-KO NMPs exhibited over 2,300 DEGs (1,264 upregulated, 1,053 downregulated) compared to WT, with mesodermal regulators up-regulated (e.g., *T*, *Tbx6*, *Msgn1*) and neural-associated genes (e.g., *Sox2*, *Hoxb9*, *Foxd3*) down-regulated (Fig. 3E). This mesodermal bias persisted till D5, with widespread activation of mesodermal genes (*Meis1*, *Meox1*) in both SC and PSM fates (Figs. 3F-H, S3B-E). Together, these observations indicate that p-Enh deletion imposes a mesoderm-primed transcriptional program as early as the NMPs stage, which expands during differentiation. Moreover, consistent with prior results (Figs. 2J-N, S3F-G), RNA-seq confirmed sustained repression of *Sox2* and upregulation of *T* in p-Enh-KO cells throughout the differentiation process. Gene ontology (GO) enrichment analyses verified the consistent activation of mesodermal genes throughout differentiation (Fig. 3I-J, S3H-I). Notably, mesodermal program was aberrantly activated in p-Enh-KO groups even under SC condition (Figs. 3J, S3I).

To investigate the activities of upstream transcription factor (TF), we performed the SCENIC analysis (Aibar et al. 2017) and then determined the TF regulon enrichment (Fig. 3K). As shown, eight distinct TF regulon modules were identified, with p-Enh-KO showing increased activity in clusters 5–8 from D3 onward. Notably, p-Enh-KO enhanced mesoderm-promoting regulons (e.g., *T*, *Tbx6*, *Cdx1* in cluster 5; *Foxf1*, *Hes1*, *Mef2c* in cluster 6; and *Meox1*, *Meis1*, *Meis2* in cluster 8) (Fig. 3L), validated by temporal profiling (Fig. S3J-O). GO analysis revealed that TF regulons responsible for somitogenesis and axial patterning are highly enriched, while neural differentiation-related TF regulons are obviously suppressed (Fig. 3M). Consistent with this transcriptional activation pattern, H3K27ac ChIP-seq in D3 NMPs revealed elevated signals at genomic loci associated with these mesoderm-promoting TF regulons in p-Enh-KO cells (Fig. S4), suggesting that epigenetic activation of lineage-specific regulatory elements may underlie the sustained upregulation of mesodermal transcriptional programs.

Together, through systematic transcriptomic analyses, we found that the transcriptional abnormalities induced by p-Enh deletion can be identified as early as the D3 NMPs stage in vitro, and the upregulation of mesodermal regulons in NMPs may drive the subsequent sustained activation of the PSM signature.

### ST-Pheno captures the spatiotemporal origin for p-Enh-KO induced T^high^Sox2^low^ NMPs subtype

**Fig. 4:**
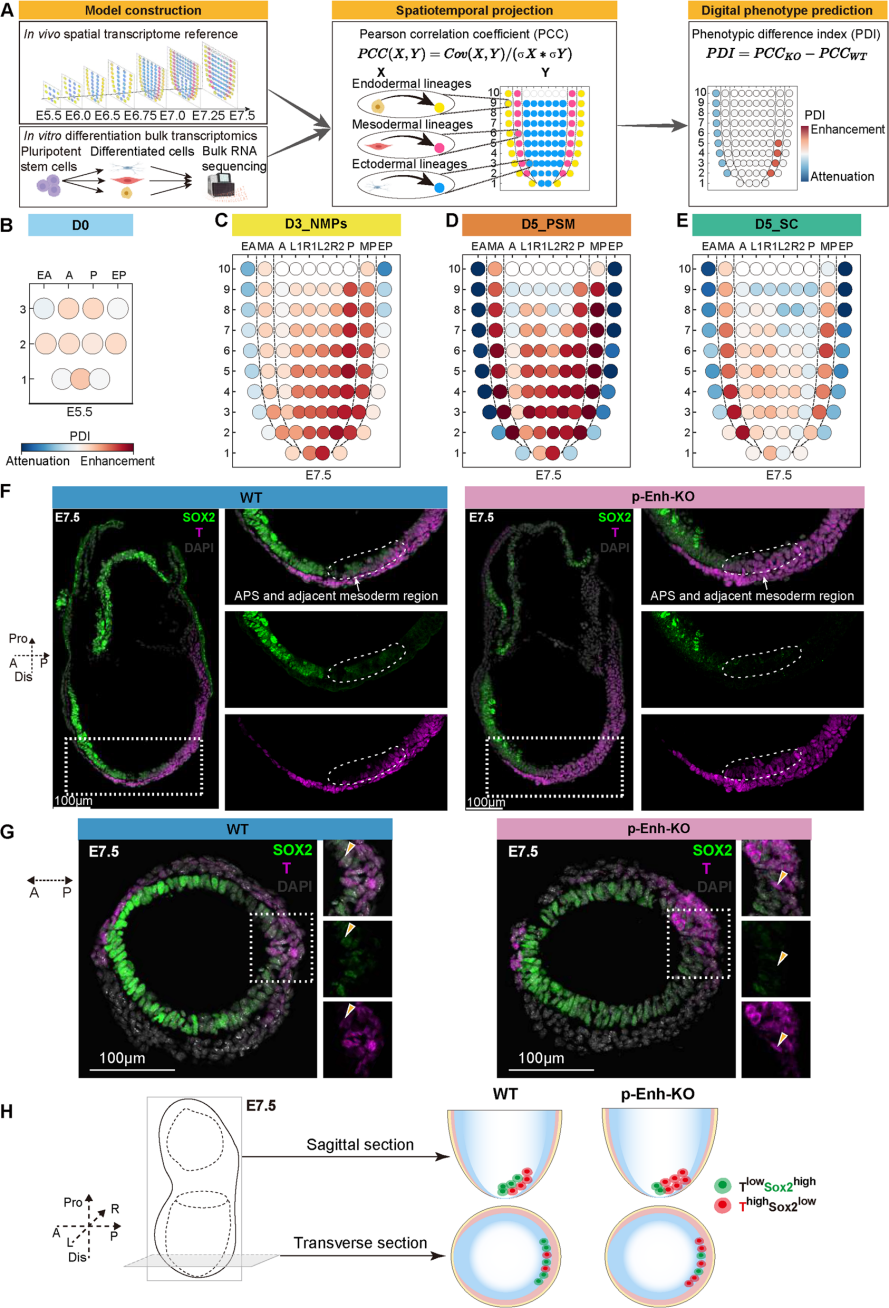
ST-Pheno maps p-Enh-KO phenotypes onto E7.5 gastrula showing in silico mesoderm-primed fate bias. **A** Workflow of the ST-Pheno framework, integrating in vivo spatiotemporal transcriptomics and in vitro differentiation data for lineage projection via Pearson correlation coefficients (PCC) analysis, and digital phenotype prediction using the phenotypic difference index (PDI). **B**-**E** Spatiotemporal PDI heatmaps comparing p-Enh-KO and WT samples across in vitro differentiation stages, digitally predicted by ST-Pheno at E5.5 or E7.5 embryonic regions. EA: Anterior endoderm; EP: Posterior endoderm; MA: Anterior mesoderm; MP: Posterior mesoderm/Primitive Streak; A/L1/R1: Anterior epiblast/ectoderm; P/L2/R2: Posterior epiblast/ectoderm. **F** Immunofluorescence of SOX2 and T in the representative sagittal sections of E7.5 WT and p-Enh-KO embryos. A: Anterior; P: Posterior; Pro: Proximal; Dis: Distal; APS: anterior primitive streak; Scale bar, 100 μm. (*n* = 8). **G** Immunofluorescence of SOX2 and T in the representative transverse sections of E7.5 WT and p-Enh-KO embryos. Yellow arrows mark double-positive cells expressing SOX2 and T. A: Anterior; P: Posterior; Scale bar, 100 μm. (*n* = 11). **H** Schematic model of NMPs subtype distribution in the distal region of E7.5 embryos. The model summarizes the altered NMPs composition in the p-Enh-KO embryo compared to WT, specifically highlighting an expansion of the mesoderm-primed (T^high^SOX2^low^) subpopulation. A, anterior; P, posterior; Pro, proximal; Dis, distal. L: left; R: right

To quantitatively determine the relevance of in vitro stem cell samples to in vivo embryo development, we developed the spatiotemporal digital phenotype prediction framework (ST-Pheno), which can project the *in vitro*-derived transcriptome onto the genuine in vivo spatial transcriptomic coordinates (Fig. 4A). Generally, ST-Pheno positions each sample at precise anatomical locations and developmental stages based on transcriptional similarity, quantified by the Pearson correlation coefficient (PCC) (see Materials and methods). Moreover, we introduced a phenotypic difference index (PDI), defined as the difference in PCC between sample groups from different conditions (e.g., genomic backgrounds or treatments), to directly quantify the transcriptomic variance across conditions (Fig. 4A). Since the PCC intrinsically measures the similarity of transcriptome profiles, the PDI effectively quantifies the change in similarity, serving as a direct measure of variance, where a larger PDI indicates greater transcriptomic divergence. Therefore, ST-Pheno enables anchoring controlled in vitro models to in vivo native context, thus enhancing the biological relevance of in vitro findings.

Here, leveraging our previously established mouse gastrula spatial transcriptome atlas (Peng et al. 2016, 2019; Wang et al. 2023), we precisely determine the spatiotemporal identities of in vitro stem cell samples undergoing PSM and SC differentiation and also pinpoint the differences between WT control and p-Enh-KO samples (Figs. 4B-E; S5A-J). To specify, ST-Pheno revealed that WT samples aligned precisely to their presumptive developmental domains, with D0 mESCs samples strongly mapping to E5.5 epiblast (Fig. S5A), D1 and D2 mESCs mapped strongly to the E7.5 epiblast (Fig. S5B), D3 NMPs localizing specifically to the posterior epiblast and primitive streak (PS) at E7.5 (Fig. S5C), D4 and D5 PSM cells mapping to E7.5 posterior mesoderm region (Fig. S5D), and D4 and D5 SC cells predominantly occupying E7.5 anterior epiblast/ectoderm (Fig. S5E). Conversely, while p-Enh-KO samples exhibited similar patterns as WT control at the mESCs stage (Figs. 4B, S5F-G, S5K-L), divergence emerged prominently at the D3 NMPs stage, with increased spatial preference towards E7.5 APS and adjacent mesoderm region (Figs. 4C, S5H). These spatiotemporal distinctions intensified over time. As shown, D4 and D5 p-Enh-KO PSM showed reinforced enrichment at mesodermal domains (Figs. 4D, S5I, M), whereas D4 and D5 p-Enh-KO SC acquired spatial coordinates overlapping mesoderm (Figs. 4E, S5J, N). Altogether, as revealed by ST-Pheno, we found that p-Enh-KO cells show distinct spatial territories compared to WT control since D3 NMPs stage, with a higher spatial preference to E7.5 APS and adjacent mesoderm region in p-Enh-KO D3 NMPs and a higher mesoderm preference for subsequent NMPs derivatives.

Finally, to validate whether the ST-Pheno predicted spatiotemporal origin of p-Enh-KO cells could indeed be detected in vivo, we carried out immunofluorescence staining for SOX2 and T on sagittal and transverse sections of E7.5 WT and p-Enh-KO embryos. As shown, we found that the APS and adjacent mesoderm region, which are marked by co-expression of SOX2 and T, in WT embryos show a pronounced accumulation of T^high^SOX2^low^ cells (Fig. 4F-G), closely aligning with our ST-Pheno predictions (Fig. 4C-E). These findings indicate that the earliest p-Enh-KO phenotypes can be traced to E7.5 APS and adjacent mesoderm region in p-Enh-KO embryos (Fig. 4H).

**Fig. 5:**
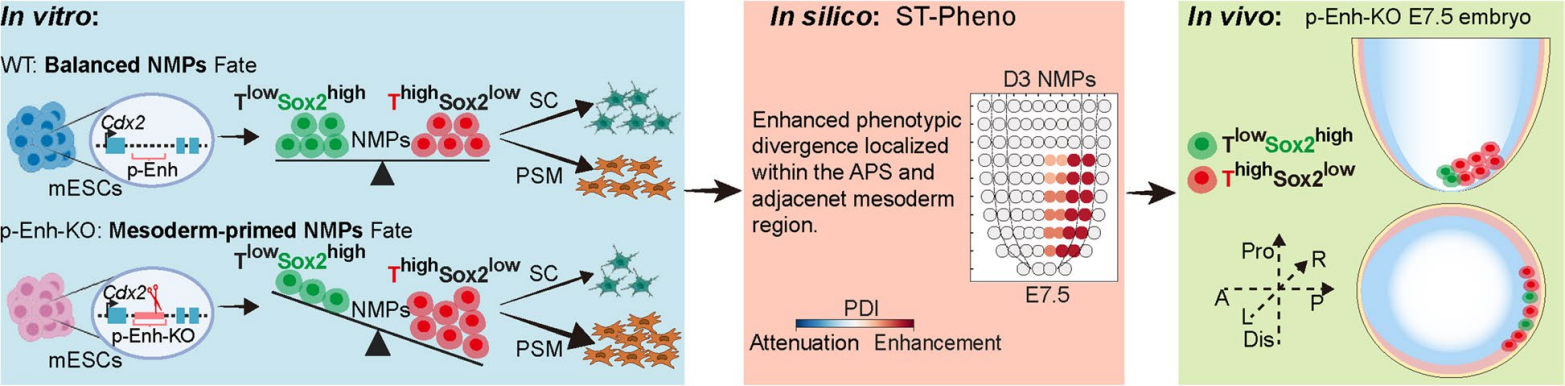
p-Enh deletion disrupts NMPs’ bi-potency and biases lineage commitment toward PSM fate across in vitro, in vivo, and in silico models. p-Enh deletion disrupts NMPs fate equilibrium, as demonstrated across in vitro differentiation, in silico ST-Pheno modeling, and *in vivo* embryogenesis: p-Enh deletion skews NMPs toward a mesoderm-primed (T^high^Sox2^low^) identity (red cells), enhancing PSM commitment while attenuating SC potential, with spatially resolved phenotypic divergence concentrated in the APS and adjacent mesoderm region (E7.5). A: Anterior; P: Posterior; Pro: Proximal; Dis: Distal; L: Left; R: Right; APS: anterior primitive streak. All schematics created with BioRender

In summary, through systematic exploration of developmental phenotypes caused by p-Enh-KO with in vitro differentiation, in silico prediction, and in vivo validation, we unraveled that p-Enh loss can lead to the dis-organization of NMPs celltype composition, and the over-produced T^high^SOX2^low^ p-Enh-KO NMPs are largely derived from E7.5 APS and adjacent mesoderm region, which may disrupt the differentiation of subsequent PSM and SC lineages (Fig. 5).

## Discussion

In this study, we systematically investigated the role of the gastrula-premarked p-Enh in early NMPs fate determination during gastrulation. Using an in vitro differentiation system, we demonstrated that loss of p-Enh disrupts the subtype compositions of NMPs, leading to the pronounced mesodermal fate bias. This bias was evident at the cellular level, marked by an increased proportion of mesoderm-primed (T^high^SOX2^low^) cells (Fig. 2), as well as at the transcriptomic level, where mesoderm-promoting TF regulons were persistently activated with the expense of neural programs (Fig. 3). Furthermore, leveraging in vivo reference and our newly developed ST-Pheno framework, we precisely mapped this fate divergence to the APS region of the E7.5 embryo, the known origin of NMPs, thereby linking our in vitro phenotype to a specific spatiotemporal coordinate in the developing embryo (Figs. 4, 5). These results highlight p-Enh's critical role in maintaining NMPs bi-potency during gastrulation.

Our current findings build upon and extend our previous research characterizing p-Enh (Chen et al. 2025), which demonstrated that p-Enh deletion reduced NMPs and PSM abundance during post-gastrulation stages (E8.5 onward), thereby defining its essential role in posterior tissue specification. We identified p-Enh's essential role as a gatekeeper in regulating NMPs fate during late gastrulation (E7.5), coinciding with its temporal activation (Yang et al. 2019). This specificity underscores p-Enh's role in maintaining NMPs bi-potency, balancing neural and mesodermal lineage potential, with increased H3K27ac at mesoderm-related genes. We propose that the later-stage reduction in NMPs and derivatives (E8.5 onward) may stem from the premature bi-potency compromise in NMPs as early as gastrulation. These insights connect enhancer function with temporal progenitor fate control, highlighting enhancer priming as crucial for developmental robustness.

Together with studies from other groups that dissected motifs within the p-Enh and their respective contributions to the regulation of nearby gene *Cdx2* (Amblard et al. 2025; Chen et al. 2025), these works collectively highlight the developmental importance of this enhancer. The role of p-Enh in early NMPs fate determination has been largely unexplored. While in vivo compensatory mechanisms may mask enhancer function, the in vitro system amplifies it, providing a unique opportunity to study p-Enh's intrinsic role in lineage specification. Given p-Enh's link to *Cdx2* regulation (Amblard et al. 2025; Chen et al. 2025), we examined whether *Cdx2* could play a role in the early NMPs fate determination process. As expected, the expression of *Cdx2* was largely compromised in p-Enh-KO cells. However, *Sox2* and *T* expression in D3 Cdx2-KO NMPs showed no significant difference from WT controls. This result further corroborates our previous notion that p-Enh can work through a *Cdx2*-independent mechanism.

While transcriptome-based GRNs analyses have identified transcription factors (e.g., *T*, *Sox2*, *Cdx2*, *Msgn1*, *Tbx6*) as key determinants of NMPs identity (Chawengsaksophak et al. 2004; Amin et al. 2016; Koch et al. 2017; Amblard et al. 2025; Braccioli et al. 2025), however, the contribution of individual regulatory elements to the wholistic regulatory network remains unresolved. Here, we demonstrate that p-Enh is indispensable for maintaining NMPs’ bi-potency and fate-balancing circuit. Its loss disrupts the *T*-*Sox2* equilibrium, increasing transcriptional heterogeneity and expanding mesoderm-primed (T^high^SOX2^low^) cells, thereby driving a profound fate bias toward PSM specification at the expense of SC development. Although the precise mechanisms by which p-Enh regulates specific key genes such as *Sox2* and *T* remain to be determined, previous study suggested that p-Enh might mediate this balance via eRNA-mediated *in trans* functions or widespread epigenetic remodeling (Fig. S4) (Chen et al. 2025). In addition, we observed a pronounced increase in H3K27ac levels at multiple mesoderm-related loci, including the *T* promoter (Fig. 2M). This finding suggests that p-Enh loss triggers broad epigenetic remodeling within NMPs, potentially involving altered activity or recruitment of H3K27 acetylation-associated writers or erasers. Such changes may give rise to enhancer competition or redistribution of chromatin-modifying machinery, thereby indirectly affecting *Sox2* regulation. Future systematic profiling of chromatin landscapes in p-Enh-KO NMPs, together with global identification of eRNA targets, will be essential to delineate the precise mechanisms linking enhancer perturbation to fate bias.

Despite the widespread use of in vitro models for studying embryogenesis and disease, standardized criteria to evaluate their fidelity to in vivo processes remain underdeveloped (Posfai et al. 2021; Kılıç and Yılmaz 2024; Yu et al. 2025). Addressing this gap, the ST-Pheno workflow we developed, provides a computational framework that leverages established spatiotemporal transcriptomic references (Peng et al. 2016, 2019; Wang et al. 2023) to project bulk in vitro transcriptomes onto in vivo developmental coordinates. ST-Pheno analysis revealed that p-Enh-KO NMPs spatially skewed toward posterior mesodermal domains, including the APS at E7.5, thereby providing in silico validation of the observed mesodermal fate bias. Beyond this application, ST-Pheno enables cost-effective spatiotemporal mapping and phenotypic comparison for diverse developmental processes-germ layer specification, enhancer perturbations, or small-molecule-driven protocols-using standard bulk RNA-seq.

Limitations include reliance on bulk RNA-seq, potentially masking NMPs heterogeneity. Future single-cell multi-omics approaches will resolve subpopulation dynamics and delineate p-Enh perturbation effects on single-cell fate trajectories.

In summary, our study uncovers a previously unrecognized early role of p-Enh in maintaining NMPs’ bi-potency and preventing mesodermal dominance during fate choice. Beyond the specific individual enhancer, our integrative experimental-computational approach opens new avenues for pinpointing the spatiotemporal consequences of cellular perturbations during the earliest steps of vertebrate body plan formation, especially at the early cell fate determination stage. Collectively, these findings establish enhancers as fundamental determinants of developmental robustness, offering mechanistic insights into congenital posterior malformations and candidate targets for regenerative medicine.

## Materials and methods

### mESCs maintenance and differentiation

mESCs were maintained under feeder-free conditions in standard 2i/LIF medium containing 3 µM CHIR99021 (Selleck Chemicals, S1263), 1 µM PD0325901 (Selleck Chemicals, S1036), and 10 ng/mL mouse leukemia inhibitory factor (LIF; Millipore, ESG1107), as previously described (Ying et al. 2008). For differentiation towards NMPs, SC, and PSM, cells were induced using a previously established protocol (Gouti et al. 2014). Briefly, 50,000 cells were plated onto Matrigel-coated 35 mm dishes in N2B27 medium supplemented with 10 ng/mL basic fibroblast growth factor (bFGF). N2B27 medium consisted of a 1:1 mixture of DMEM/F12 and Neurobasal medium (GIBCO), further supplemented with 1 × N2 supplement (GIBCO), 1 × B27 supplement (GIBCO), 1 × GlutaMAX (GIBCO), 40 mg/mL bovine serum albumin (BSA; Roche), 100 U/mL penicillin, 100 µg/mL streptomycin, and 0.1 mM 2-mercaptoethanol. To specify NMPs fate, cultures were exposed to 5 µM CHIR99021 between 24 to 36 h post-seeding. For SC differentiation, the Smoothened agonist SAG (Selleck Chemicals, S7779) was added from day 3 (D3) to D5. PSM induction was achieved by continuous treatment with 5 µM CHIR99021 from D3 to D5.

### RNA extraction, Quantitative real-time PCR (qPCR) and RNA sequencing library preparation

Total RNA was isolated from in vitro-cultured cells using TRIzol Reagent (Invitrogen, cat # 15,596,018) according to the manufacturer's instructions. cDNA was synthesized from purified RNA using FastQuant RT Super Mix (Tiangen, cat #KR108), following the supplier's protocol. qPCR was performed using diluted cDNA template (1:10), Stormstar SYBR Green qPCR Master Mix (DBI Bioscience, cat #DBI-143), and gene-specific primers (Table S1). For RNA sequencing (RNA-seq), libraries were prepared from 300 ng of high-quality total RNA with the NEBNext Ultra II RNA Library Prep Kit for Illumina (NEB, cat #E7775L). The resulting libraries underwent paired-end sequencing (150 bp read length) on an Illumina NovaSeq 6000 platform.

### Immunofluorescence staining

Immunofluorescence was performed as previously described (Im et al. 2018). For cultured cells, samples were rinsed once with phosphate-buffered saline (PBS), fixed in 4% paraformaldehyde (PFA)/PBS (pH 7.3) for 30 min at room temperature (RT), then permeabilized and blocked in PBS containing 0.5% Triton X-100 and 5% bovine serum albumin (BSA) for 1 h. Embryos were fixed overnight at 4 °C in 4% PFA/PBS, cryoprotected through a graded sucrose series (10%, 20%, 30% w/v), embedded in optimal cutting temperature (OCT) compound (Tissue-Tek), and cryosectioned at 16 μm using a Leica CM3050S cryostat. Both sample types underwent subsequent immunostaining under identical conditions: incubation with primary antibodies diluted in blocking buffer overnight at 4 °C (Table S2), followed by three washes in PBS containing 0.1% Triton X-100 (PBS-Tri), incubation with secondary antibodies (1:400 in PBS-Tri) for 2 h at RT, and final washes. Nuclei were counterstained with DAPI prior to coverslip mounting. Cultured cells were imaged on a Leica TCS SP8 STED confocal microscope, while tissue sections were imaged using an Olympus FV3000 confocal microscope. All images were segmented using Cellpose (Stringer et al. 2020) and fluorescence intensity quantified with ImageJ software (Schroeder et al. 2020).

### Generation of p-Enh-KO mESCs

Genomic knockout of specific fragments in mESCs and mice was performed as previously described (Ran et al. 2013). Briefly, single-guide RNAs (sgRNAs) targeting enhancer region were designed using the Chop-Chop web tool (http://chopchop.cbu.uib.no/). Complementary sgRNA oligonucleotides were annealed and cloned into linearized px330-mCherry vector (Addgene #98,750). The resulting px330-mCherry-sgRNA plasmid was transfected into E14TG2a mESCs using Lipofectamine 3000 (Invitrogen, L3000008) per manufacturer's instructions. At 48 h post-transfection, mCherry-positive cells were isolated via fluorescence-activated cell sorting (FACS) using a BD FACS Aria SORP and cultured for 4 to 5 days to permit clonal expansion. Individual colonies were manually picked for expansion, followed by genotyping and Sanger sequencing verification. Potential off-target sites were computationally predicted using Chop-Chop analysis. All sgRNA designs and genotyping primers are detailed in Table S3.

### Animals, embryo staging, and collection

All animal procedures were conducted in accordance with protocols approved by the Institutional Animal Care and Use Committee of Guangzhou National Laboratory (Protocol No. GZLAB-AUCP-2022–10-A04) and performed at the institution's Animal Core Facility. Timed matings were established between sexually mature (≥ 6 weeks old) wild-type C57BL/6 mice and p-Enh-KO mice (Chen et al. 2025). Embryonic day 0.5 (E0.5) was designated upon detection of vaginal plugs. At desired embryonic stages, pregnant females were euthanized and embryos harvested. Embryos were dissected from uterine tissues under an Olympus stereomicroscope, transferred to PBS-filled dishes, and manually cleared of extra-embryonic tissues. Developmental staging and morphological assessment were performed according to Theiler's criteria. Processed embryos were immediately utilized in subsequent analyses.

### RNA-seq data processing

Raw sequencing reads were subjected to adapter and quality trimming using Trim Galore (v0.4.4_dev) (Martin 2011). Cleaned reads were subsequently aligned to the mouse reference genome (mm10) using STAR (v2.5.2b) with default parameters (Dobin et al. 2012). Duplicate reads were removed to minimize PCR bias, and uniquely mapped reads were quantified at the gene level using featureCounts (v1.6.5) (Liao et al. 2013). Gene annotations were derived from the GENCODE vM10 comprehensive gene annotation set (gencode.vM10.annotation.gtf).

### Principal component and hierarchical clustering analyses

Raw count matrices were transformed using the variance-stabilizing transformation (VST) function implemented in DESeq2 (Love et al. 2014). The top 2,000 most variable genes were selected based on VST-normalized expression values and used for PCA and hierarchical clustering.

### Differential expression analysis

Raw gene expression counts were normalized using DESeq2's median-of-ratios method to account for library size differences between samples. Differential expression analysis was performed by using DESeq2 and significantly DEGs were defined as the genes with adjusted *p*-value less than 0.01 and fold change greater than 1.5 (Love et al. 2014).

### Gene Ontology (GO) enrichment analysis

To identify biological processes associated with transcriptional changes, GO enrichment analysis was performed using the clusterProfiler package (v4.8.2) in R (Wu et al. 2021). Gene Ontology annotations were retrieved from the Gene Ontology Consortium database (http://geneontology.org). Enriched GO terms were ranked based on adjusted *p*-value calculated via the Benjamini–Hochberg procedure.

### Transcription factor regulon analysis

Transcription factor regulon inference was conducted using the scenicpy implementation of the SCENIC workflow (Aibar et al. 2017). First, co-expression modules were identified by applying the GRNBoost2 algorithm to the normalized bulk expression matrix (cells x genes) with default settings; TF-target gene pairs scoring above 0.001 were retained for further analysis. Next, putative regulons were refined via motif enrichment: for each candidate TF module, cis target scanned regulatory regions (± 10 kb around the transcription start site) for overrepresented DNA-binding motifs corresponding to the TF of interest. Finally, regulon activity was quantified on a per-cell basis using AUCell, and only regulons exhibiting a maximum activity score greater than 0.05 across all cells were carried forward. To reveal patterns of coordinated regulatory activity, the filtered regulon activity matrix was clustered by k-means using the scikit-learn package. The list of inferred All immunostaining and quantitative experiments for WT and p-Enh-KO cells or mice were performed in at least three independent biological replicates. Data are presented as mean ± standard error of the mean (SEM). Statistical analyses were conducted using GraphPad Prism version 8.0.0. Comparisons between two groups were made with an unpaired, two-tailed Students t-test. A *p*-value < 0.05 was considered statistically significant.

RNA-seq count data were processed and normalized using DESeq2 (Love et al. 2014). Briefly, sample-specific size factors were computed as the median of gene-wise count ratios relative to the geometric mean across all samples, thereby correcting for differences in sequencing depth. Differential expression was then assessed within a negative binomial generalized linear model framework: first, gene-specific dispersion estimates were obtained and moderated via empirical Bayes shrinkage to stabilize variance estimates; next, the GLM was fitted to model treatment- vs. control-associated changes in expression; and finally, Wald tests were applied to each coefficient to evaluate significance. Resulting *p*-value were adjusted for multiple comparisons using the Benjamini–Hochberg procedure to control the false discovery rate.

Gene Ontology enrichment analysis was carried out with the clusterProfiler package (Wu et al. 2021). Overrepresentation of GO terms among differentially expressed genes was determined by a one-sided hypergeometric test, with subsequent correction for multiple testing again performed using the Benjamini–Hochberg method to identify the most robustly enriched biological processes.

### Spatiotemporal digital phenotype prediction (ST-Pheno)

To quantitatively compare spatiotemporal transcriptional phenotypes between cell lines, we developed the ST-Pheno framework. Expression profiles were harmonized to enable direct cross-dataset comparison: Transcript abundances from a published Geo-seq dataset (Peng et al. 2016, 2019; Wang et al. 2023), serving as a spatiotemporally resolved reference atlas for embryonic region, were converted to Log_2_(TPM + 1). Bulk RNA-seq samples generated from the KO and WT cell lines underwent an equivalent normalization process; raw read counts were first normalized to transcripts per million (TPM), followed by the Log_2_(TPM + 1) transformation. To specifically capture spatially informative transcriptional variation, downstream ST-Pheno analysis focused exclusively on the set of DEGs previously defined by Peng et al. as characteristic markers of discrete spatial domains within the reference embryo (Peng et al. 2019), thereby restricting the analysis to genes exhibiting known spatiotemporal domain-specific expression patterns.

Spatiotemporal projection of in vitro cellular states onto the embryonic reference atlas was achieved by quantifying pairwise transcriptome similarity. For each bulk RNA-seq sample and each spatial domain profile within the Geo-seq atlas, the Pearson Correlation Coefficient (PCC) was calculated as $\mathit{PCC}(X,Y)=Cov(X,Y)/(\sigma X*\sigma Y)$, where *X* represents a bulk sample vector, *Y* represents a Geo-seq spatial domain vector, *Cov* is the covariance, and *σ* denotes the standard deviation. The resulting PCC value provides a quantitative measure of the concordance between a bulk sample's transcriptome and the characteristic gene expression signature defining a specific spatial domain, thereby assigning each bulk sample a spatial resemblance score across the embryonic landscape. For each bulk RNA-seq sample, the spatial domain(s) exhibiting the highest PCC value(s) represent the inferred embryonic location(s) whose characteristic expression pattern its global transcriptome most closely resembles, with higher PCC values indicating greater similarity; this PCC matrix serves as the basis for spatial mapping within the reference system.

To systematically identify and quantify the spatiotemporal phenotypic differences between KO and WT cell lines predicted by the PCC mapping, a phenotypic difference index (PDI) was derived. For each corresponding spatial domain mapped by PCC, the PDI was calculated as the difference between the PCC value of the KO sample and that of the WT sample: $PDI={PCC}_{KO}-{PCC}_{WT}$ The PDI, ranging from −2 to 2, serves as a quantitative indicator of the relative change in the cell line's transcriptional mimicry of each embryonic domain due to the genetic perturbation: a PDI of 0 indicates no observable change in association strength relative to the spatial domain; values ranging from 0 to 2 signify an enhancement in the KO sample’s transcriptional concordance compared to WT; conversely, values ranging from −2 to 0 signify an attenuation in the KO sample’s transcriptional concordance compared to WT. This index thus provides a direct computational prediction of the spatiotemporal phenotypic shift resulting from the knockout.

### Calculating correlation between in vitro bulk RNA-seq data and in vivo mouse embryo single-cell RNA-seq data

We obtained the processed data of in vivo mouse embryo single-cell RNA-seq data, cell type annotation information and the marker gene list from https://github.com/MarioniLab/EmbryoTimecourse2018. Then, we generated pseudo-bulk samples of cell types and merged the top 50 marker genes of cell types as the feature gene set for calculating Spearman’s correlation coefficients between in vitro samples and in vivo pseudo-bulk samples.

### H3K27ac ChIP-seq data processing

We initiated the analysis of ChIP-seq samples starting from raw sequencing reads. Firstly, Trim Galore (version 0.4.4_dev) was used to remove adapter and low-quality sequences by trimming 3’ ends of reads. The resulting reads were then aligned to mouse reference genome mm10 using Bowtie (version 1.2.2) with the parameters “–chunkmbs = 512 -I = 0 -X = 1000 –best -m = 1”. After removing duplicates, we performed peak calling using MACS (version 1.4.2) with the parameters “–shiftsize = 100 –nomodel –keep-dup = all”. Subsequently, we merged all resulting peaks of samples into a consensus list of genomic regions and counted the reads within those regions using MAnorm2_utils (version 1.0.0) with the parameters “–min-peak-gap = 150 –typical-bin-size = 2000 –shiftsize = 100 –filter = blacklist”. The blacklist regions of mm10 were obtained from Amemiya et al. (Amemiya et al. 2019).

### Identification of differential H3K27ac regions

We used MAnorm2 (version 1.2.2) to identify differential H3K27ac regions between WT and p-Enh-KO D3 NMPs samples with the cutoffs adjusted *p*-value < 0.05. Distal regulatory elements were defined as the H3K27ac regions located greater than 1.5 kb from transcription start sites (TSSs) of genes. The gene annotation file of mm10 was obtained from the GENCODE project.

### Statistical analysis

All immunostaining and quantitative experiments for cells and embryos of both WT and p-Enh-KO genotypes were performed in at least three independent biological replicates. Data are presented as mean ± standard error of the mean (SEM). Statistical analyses were conducted using GraphPad Prism version 8.0.0. Comparisons between two groups were made with an unpaired, two-tailed Students t-test. A *p*-value < 0.05 was considered statistically significant.

RNA-seq count data were processed and normalized using DESeq2 (Love et al. 2014). Briefly, sample-specific size factors were computed as the median of gene-wise count ratios relative to the geometric mean across all samples, thereby correcting for differences in sequencing depth. Differential expression was then assessed within a negative binomial generalized linear model framework: first, gene-specific dispersion estimates were obtained and moderated via empirical Bayes shrinkage to stabilize variance estimates; next, the GLM was fitted to model treatment- vs. control-associated changes in expression; and finally, Wald tests were applied to each coefficient to evaluate significance. Resulting *p*-value were adjusted for multiple comparisons using the Benjamini–Hochberg procedure to control the false discovery rate.

Gene Ontology enrichment analysis was carried out with the clusterProfiler package (Wu et al. 2021). Overrepresentation of GO terms among differentially expressed genes was determined by a one-sided hypergeometric test, with subsequent correction for multiple testing again performed using the Benjamini–Hochberg method to identify the most robustly enriched biological processes.

Analysis of H3K27ac ChIP-seq data for WT and p-Enh-KO D3 NMPs samples, each condition with two biological replicates, was performed by MAnorm2 (Tu et al. 2020). For normalization of ChIP-seq signals, MAnorm2 firstly identified common peaks between samples as baseline regions. These common peaks are then subjected to MA-plot (log-ratio vs. average signal intensity), where a linear regression model is fitted to calculate sample-specific scaling factors. Finally, these scaling factors are applied to adjust the ChIP-seq signals across all peaks, including sample-specific peaks. For differential analysis, MAnorm2 employs a modified t-test based on both the empirical Bayes framework to improve variance estimation for small sample sizes and the mean–variance modeling to stabilize variance estimates by borrowing information across genomic regions. *P*-values were corrected for multiple testing by using Benjamini-Hochberg (BH) method.

## Supplementary Information

Supplementary Material 1: Figure S1. Validation of p-Enh-KO cell lines, image-based cell identification, and characterization of promoter H3K27ac and gene expression changes. Figure S2. Correlation of daily time-course bulk RNA-seq from WT and p-Enh-KO. Figure S3. Transcriptomic alterations in p-Enh-KO vs. WT cells towards PSM and SC lineages. Figure S4. Increased H3K27ac enrichment at mesodermal genes loci in D3 p-Enh-KO NMPs. Figure S5. Spatiotemporal correlation analysis of in vitro differentiation trajectories mapped to embryonic reference.

Supplementary Material 2: Table S1. List of quantitative PCR primer sequences. Table S2. List of primary antibodies and their applications. Table S3. Target sequences of sgRNAs for generating p-Enh-KO cell lines.

## Data Availability

The raw sequence data of bulk RNA-seq reported in this paper have been deposited in the Genome Sequence Archive (Chen et al. 2021) in National Genomics Data Center (CNCB-NGDC Members and Partners 2024), China National Center for Bioinformation/Beijing Institute of Genomics, Chinese Academy of Sciences (GSA: CRA029068) that are publicly accessible at https://ngdc.cncb.ac.cn/gsa. Geo-seq data used in this paper comes from the public data GEO: GSE120963. H3K27ac ChIP-seq data from D3 NMPs were obtained from GSA: CRA014616. All other data are available from the corresponding authors upon request.

## Abbreviations

p-Enh: Posterior enhancer
NMPs: Neuromesodermal progenitors
SC: Spinal cord
PSM: Presomitic mesoderm
ST-Pheno: Spatiotemporal Phenotype prediction framework
D: Day
E: Embryonic day
APS: Anterior primitive streak
GRNs: Gene regulatory networks
WNT: Wingless/Integration signaling pathway
FGF: Fibroblast Growth Factor signaling pathway
RA: Retinoic acid
KO: Knockout
WT: Wild-type
mESCs: Mouse embryonic stem cells
DEGs: Differentially expressed genes
PCA: Principal component analysis
GO: Gene Ontology
TF: Transcription factor
PCC: Pearson correlation coefficient
PDI: Phenotypic difference index
PS: Primitive streak
qPCR: Quantitative Polymerase Chain Reaction
sgRNA: Single-guide RNA
ChIP-seq: Chromatin Immunoprecipitation followed by sequencing
RNA-seq: RNA sequencing
SCENIC: Single-Cell rEgulatory Network Inference and Clustering
eRNA: Enhancer RNA
