# Supplementary material for: Posterior enhancer (p-Enh) maintains early neuromesodermal progenitors bi-potency during gastrulation
Source: Cell Regen. 2025 Nov 15;14:44. doi: 10.1186/s13619-025-00272-8 (PMC12618760; doi:10.1186/s13619-025-00272-8)
Supplement: Supplementary file 1 — Supplementary Material 1: Figure S1. Validation of p-Enh-KO cell lines, image-based cell identification, and characterization of promoter H3K27ac and gene expression changes. Figure S2. Correlation of daily time-course bulk RNA-seq from WT and p-Enh-KO. Figure S3. Transcriptomic alterations in p-Enh-KO vs. WT cells towards PSM and SC lineages. Figure S4. Increased H3K27ac enrichment at mesodermal genes loci in D3 p-Enh-KO NMPs. Figure S5. Spatiotemporal correlation analysis of in vitro differentiation trajectories mapped to embryonic reference. [file 13619_2025_272_MOESM1_ESM.pdf]

Supplementary Figure 1

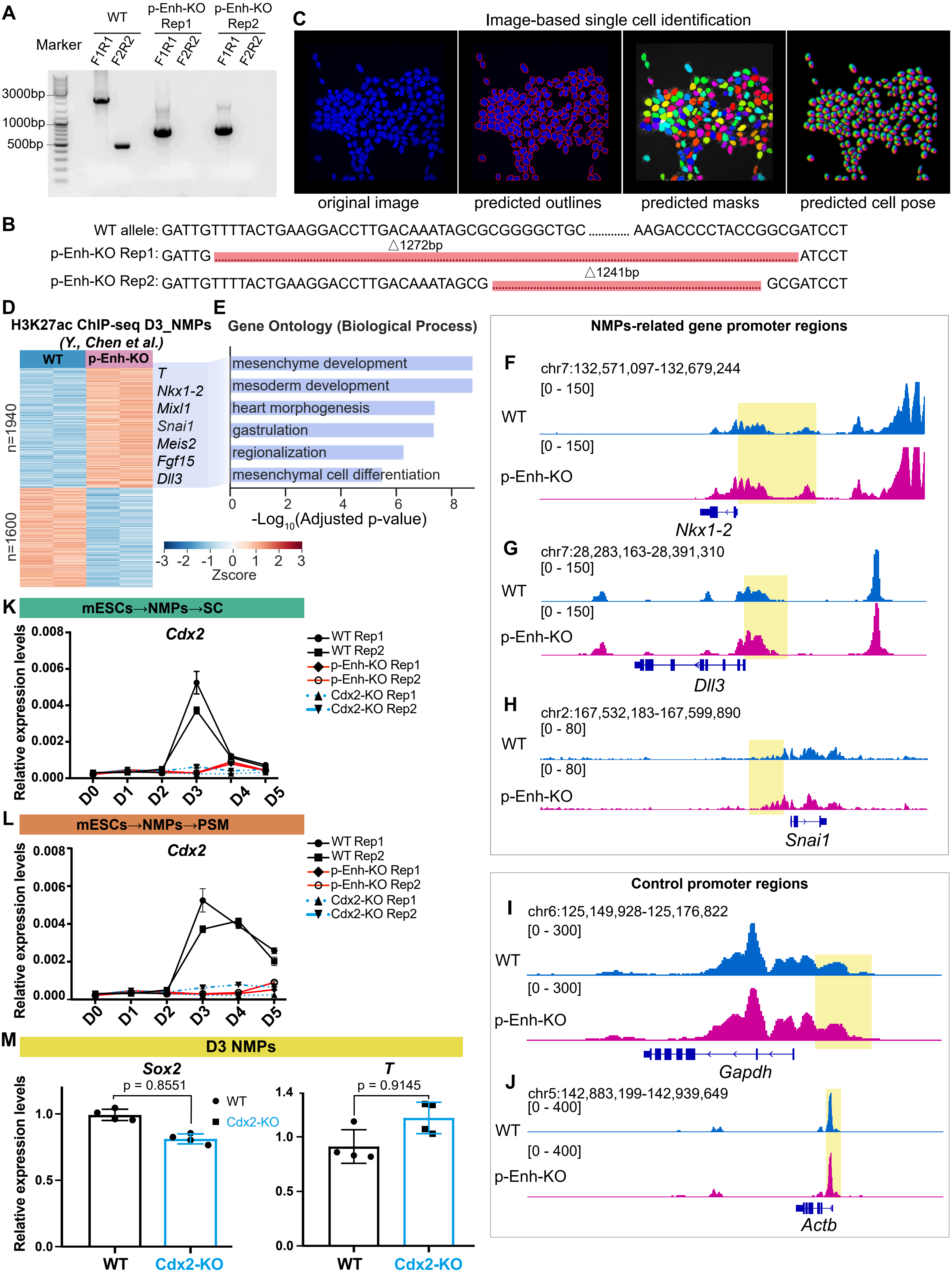

Supplementary Figure 2
Panel 1

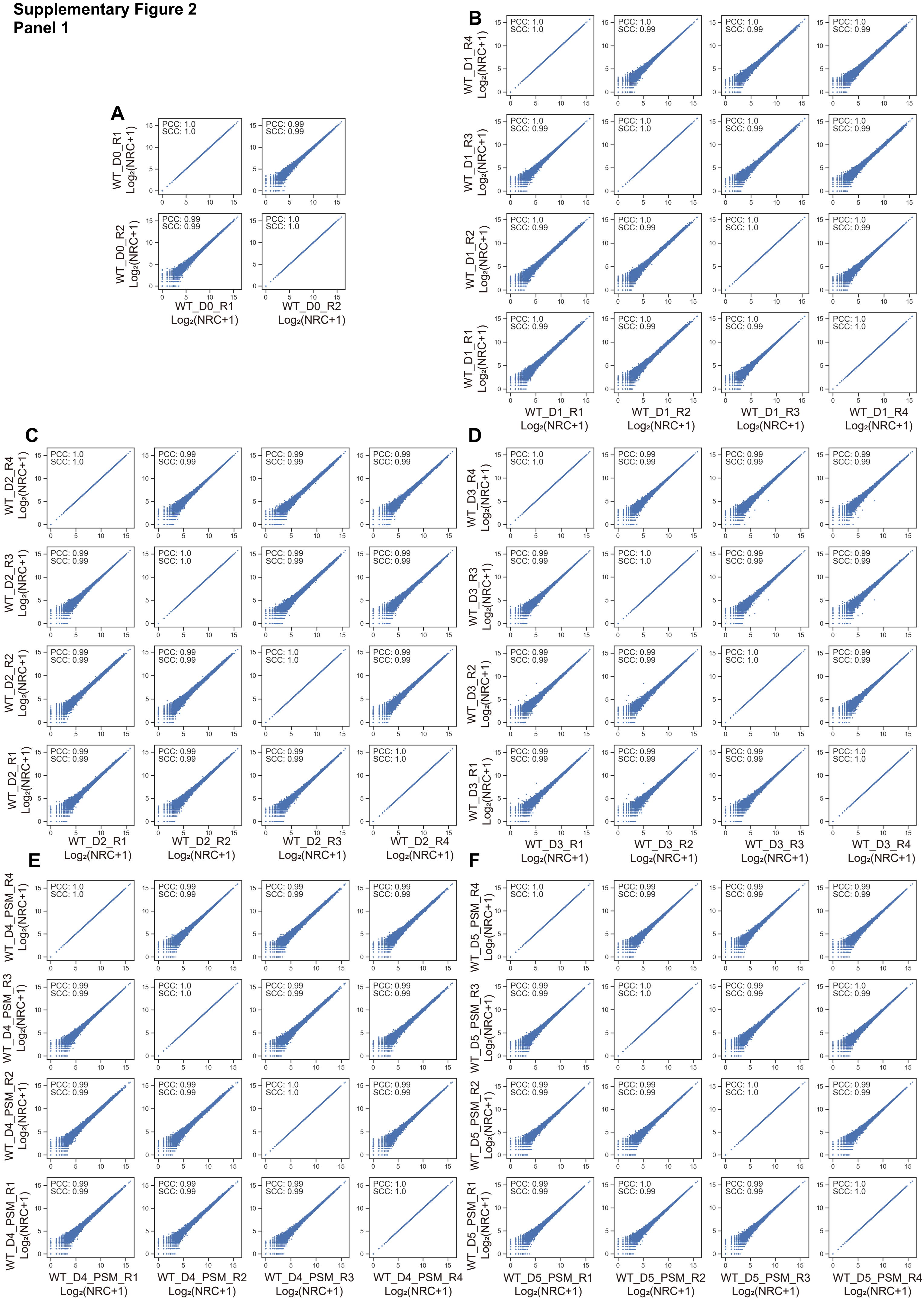

Supplementary Figure 2
Panel 2

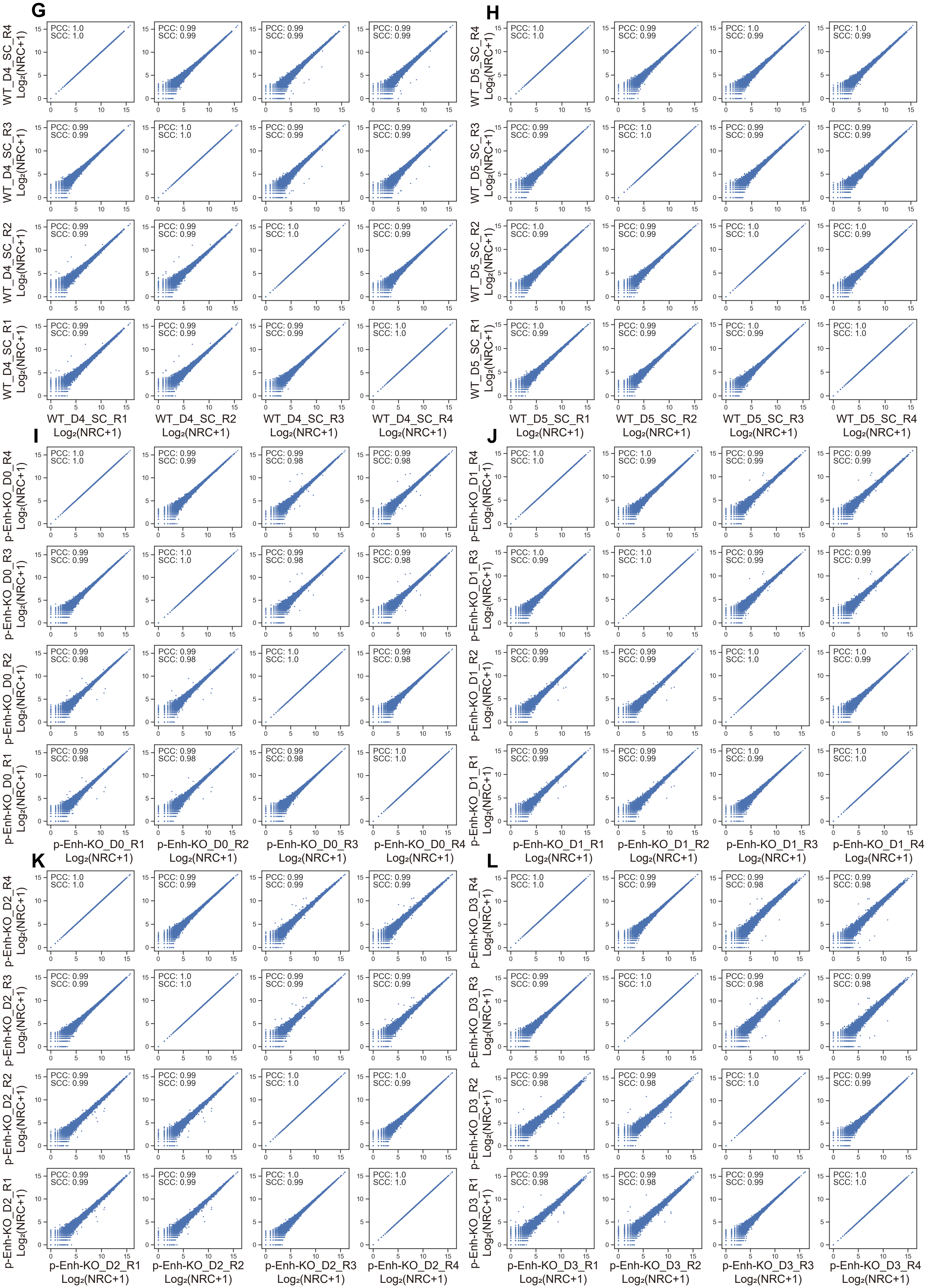

Supplementary Figure 2
Panel 3

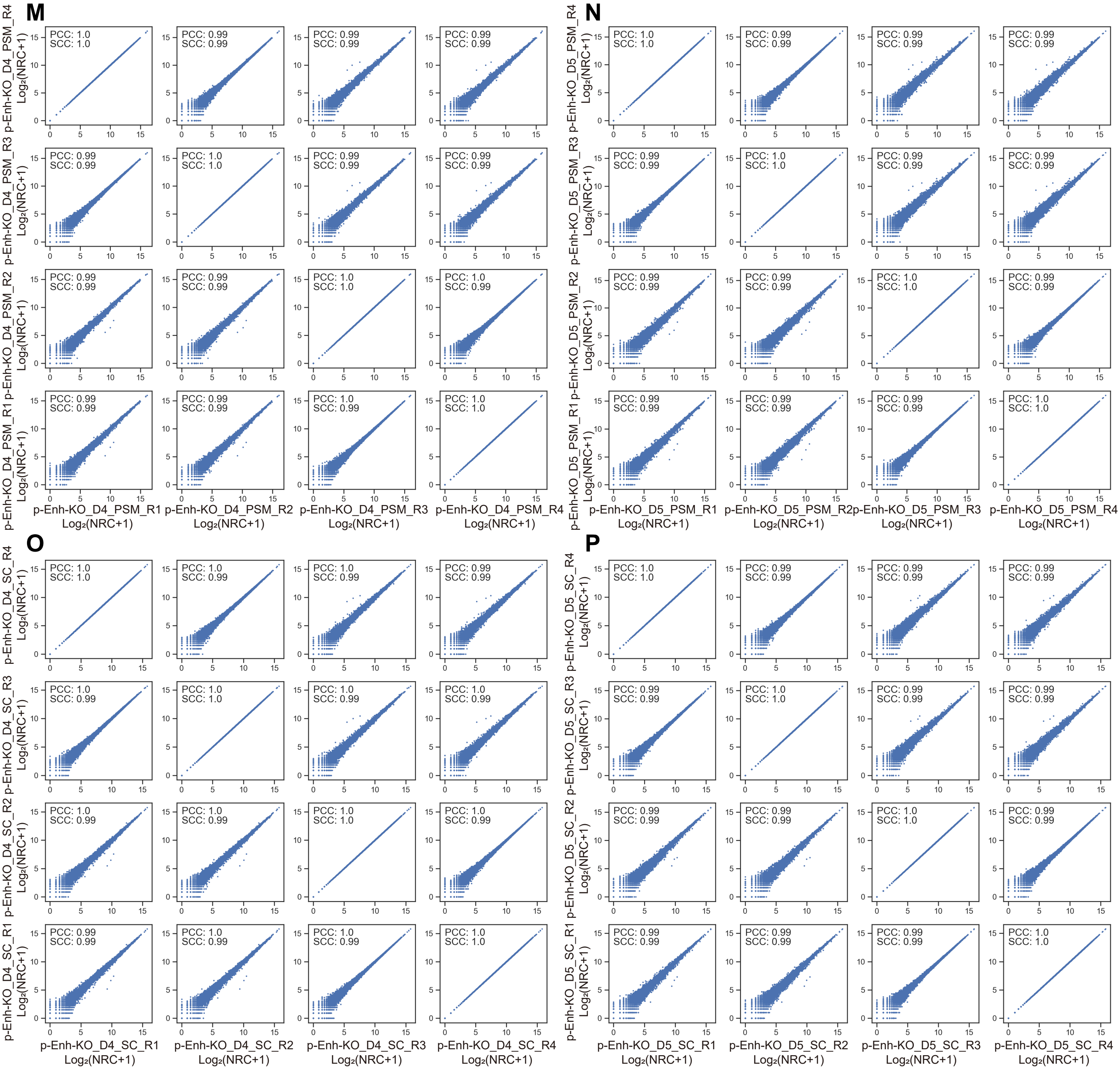

Supplementary Figure 3

Panel 1

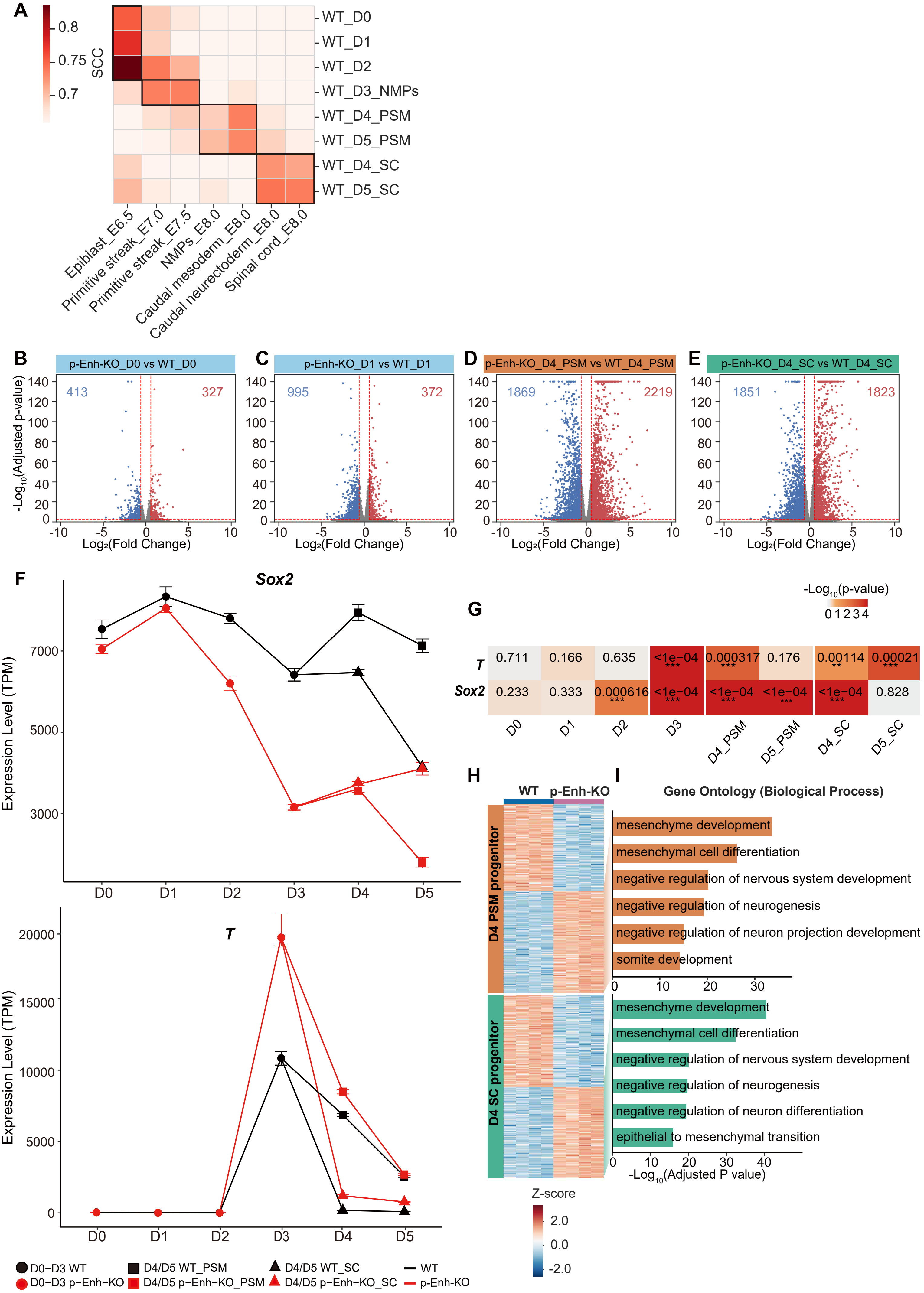

Supplementary Figure 3
Panel 2

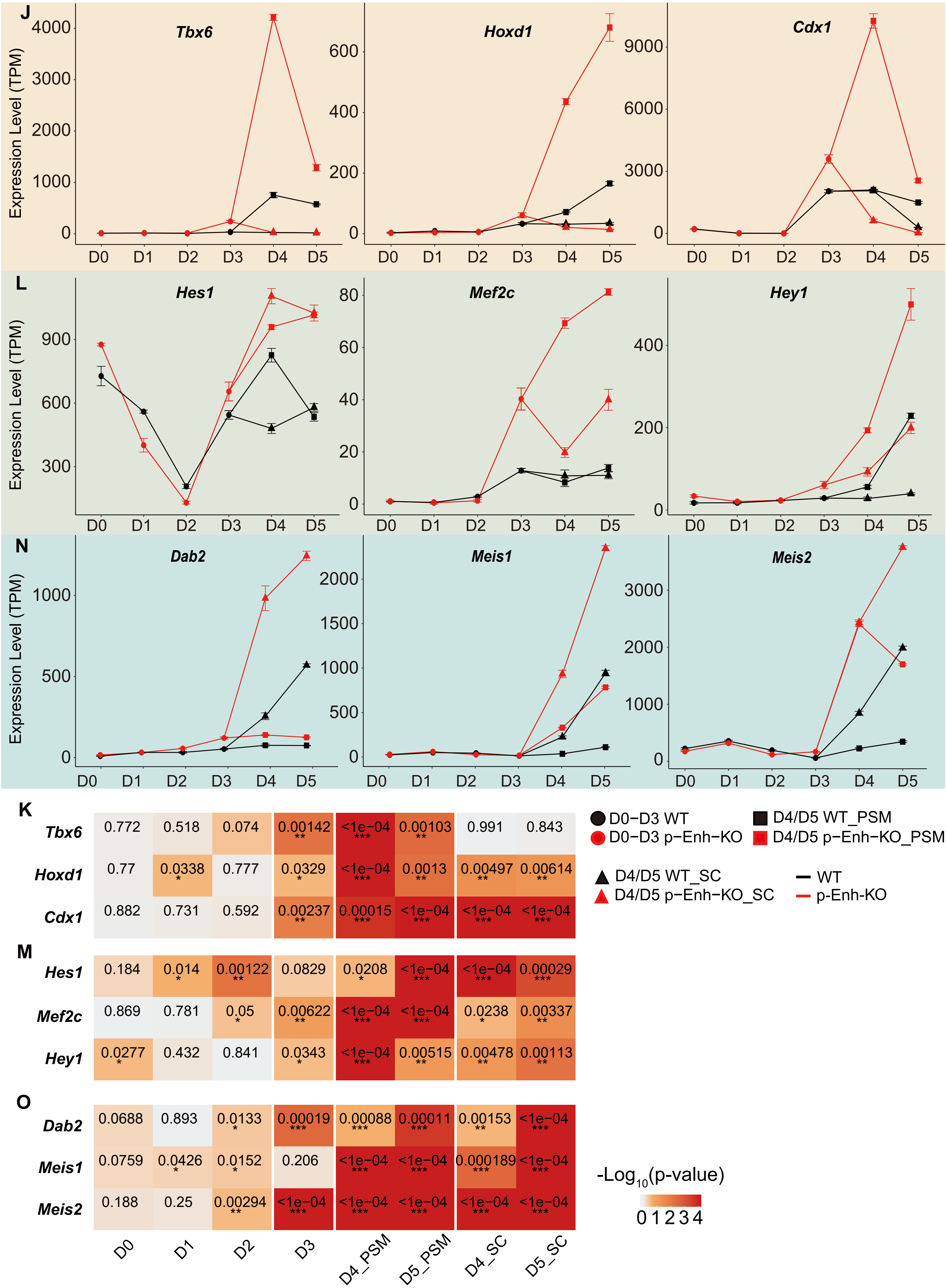

Supplementary Figure 4

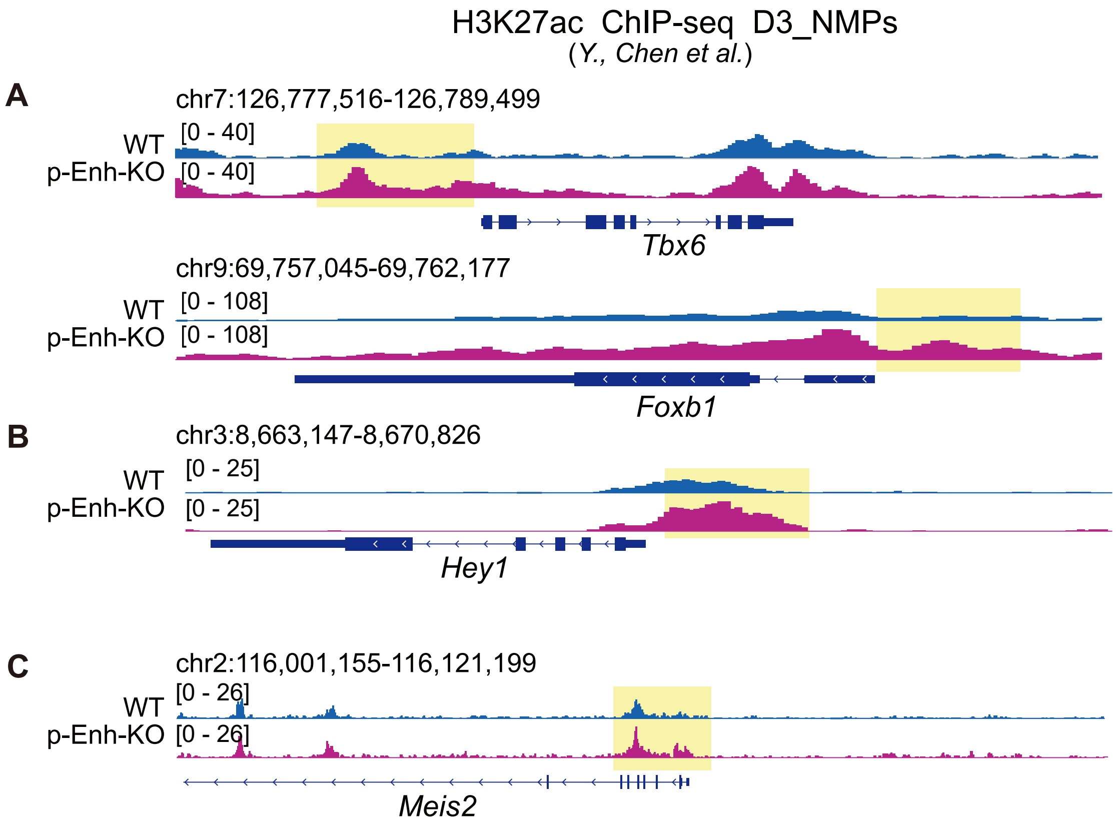

Supplementary Figure 5

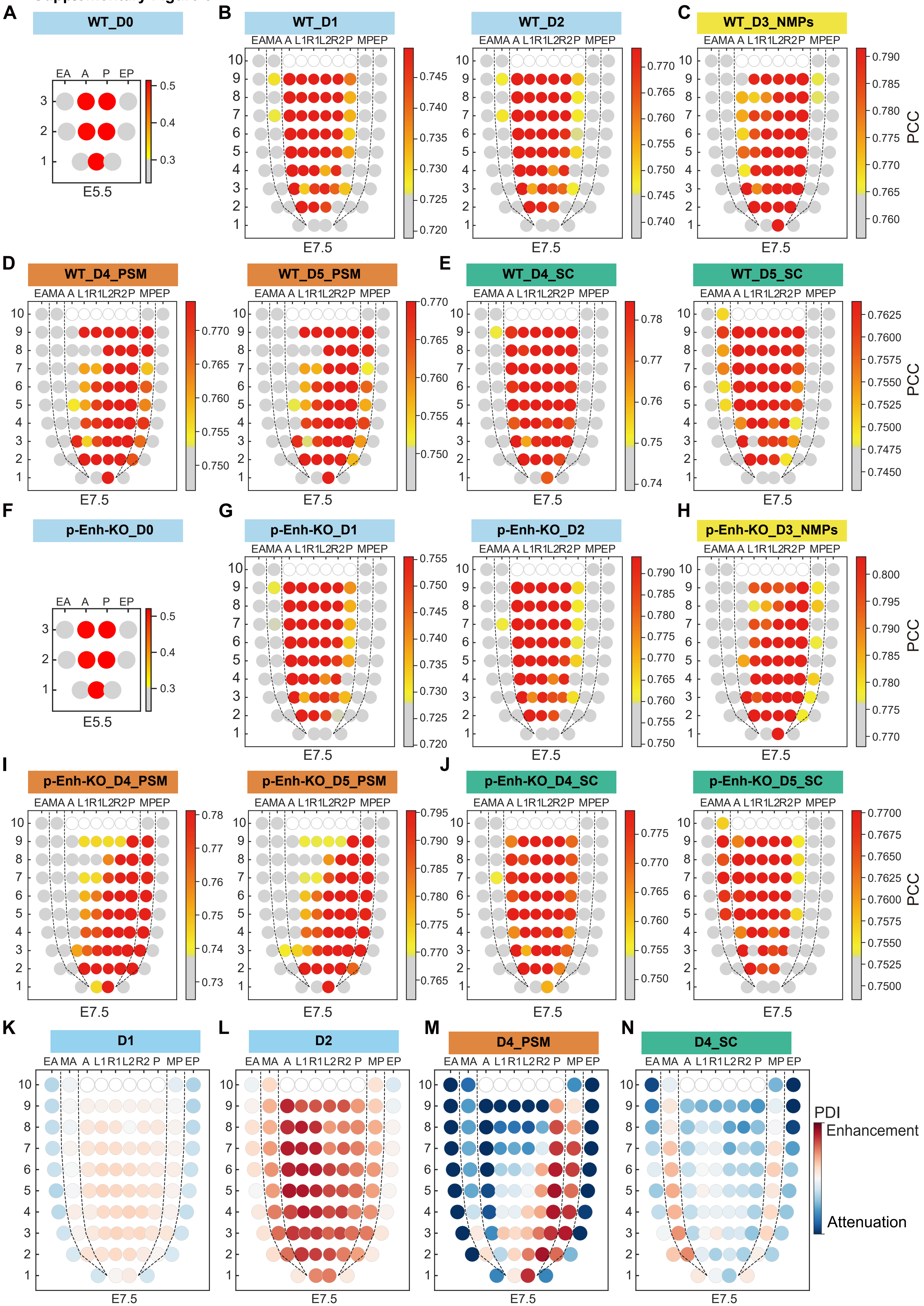

Supplementary Figure legends

Fig.S1. Validation of p-Enh-KO cell lines, image-based cell identification, and characterization of promoter H3K27ac and gene expression changes.

A: Genotyping results for the WT and p-Enh-KO cell lines. Primers used for genotyping were listed in Table S3.
B: Sanger sequencing confirming deletion region in p-Enh-KO cell lines vs. WT. Deletion sizes are 1272 bp (Rep1) and 1241 bp (Rep2), respectively.
C: Image-based single-cell identification workflow used for quantifying DAPI-positive cells and delineating individual cell boundaries in each field of view. Cell segmentation and identification were performed using the Cellpose software.
D: Heatmap of H3K27ac ChIP-seq signals at promoter regions in D3 NMPs (WT vs. p-Enh-KO). p-Enh-KO results in a widespread gain of H3K27ac at promoters of 1940 genes (Chen et al., 2025).
E: GO enrichment analysis of the 1,940 genes showing increased promoter H3K27ac in p-Enh-KO cells (as shown in **D**) reveals strong enrichment for biological processes related to mesodermal lineage specification and tissue morphogenesis.
F-H: IGV snapshots showing representative promoter regions of mesodermal and NMPs-related genes (*Nkx1-2*, *Dll3*, and *Snai1*) with increased H3K27ac signals in p-Enh-KO cells. Promoter regions are highlighted in yellow; genomic coordinates are shown below.
I-J: Representative promoter regions of housekeeping genes (*Gapdh* and *Actb*) show no notable change in H3K27ac levels between WT and p-Enh-KO. Promoter regions are highlighted in yellow; genomic coordinates are shown below.
K-L: Temporal expression profiling of *Cdx2* in WT, p-Enh-KO, and Cdx2-KO cells during *in vitro* differentiation (days 0-5), analyzed by qPCR (n=2).
M: qPCR analysis of NMPs markers (*T* and *Sox2*) expression at D3 NMPs in WT versus Cdx2-KO cells (n=4).

Fig.S2. Correlation of daily time-course bulk RNA-seq from WT and p-Enh-KO.

A-P: Scatter plots showing correlation between biological replicates of transcriptomes. Plots correspond to: WT (**A-H**), p-Enh-KO (**I-P**). All samples have two biological replicates, where each biological replicate includes two technical replicates. Both Pearson (PCC) and Spearman (SCC) correlation coefficients are displayed.

Fig.S3. Transcriptomic alterations in p-Enh-KO vs. WT cells towards PSM and SC lineages.

A: Spearman correlation coefficients (SCC) were calculated between bulk RNA-seq data from WT samples at different days of differentiation (D0–D5) and publicly available single-cell RNA-seq data from key embryonic tissues (Pijuan-Sala et al., 2019).
B-E: Volcano plots of DEGs in p-Enh-KO vs. WT at Day 0 (**B**), Day 1 (**C**), Day 4 PSM (**D**) and Day 4 SC (**E**). Genes significantly upregulated (red) or downregulated (blue) in p-Enh-KO are shown. DEGs counts are shown in blue/red.
F: Temporal expression profiling of *T* and *Sox2* in WT (black) vs p-Enh-KO (red) during differentiation toward PSM (circles) and SC (triangles).
G: Heatmap representation of -Log_{10}(p-value) for differential expression of *T* and *Sox2* in p-Enh-KO vs. WT at each time point. p-value below 1e-04 are displayed as <1e-04.
H: Heatmaps of DEGs in WT vs. p-Enh-KO cells at two developmental stages (D4 PSM and D4 SC). Gene expression values were normalized with Z-score across all samples.
I: GO enrichment analysis for genes upregulated in p-Enh-KO relative to WT at the stages shown in (**H**). Bars represent the -Log_{10}(adjusted p-value) for each enriched term.
J-O: Temporal RNA-seq profiling of regulons activities for clusters 5 (**I**), 6 (**K**) and 8 (**M**) (related to Fig. 3K and 3L) in WT (black) vs. p-Enh-KO (red) cells during differentiation toward PSM (circles) and SC (triangles). Heatmaps showing -Log_{10}(p-value) for differential regulon activities in (**I**), (**K**) and (**M**) comparing p-Enh-KO to WT at each time point. p-value below 1e-04 are displayed as <1e-04.

Fig.S4. Increased H3K27ac enrichment at mesodermal genes loci in D3 p-Enh-KO NMPs.

A-C: IGV snapshots showing H3K27ac ChIP-seq profiles at representative loci from clusters 5 (**A**), 6 (**B**) and 8 (**C**) (related to Fig. 3K and 3L) (Chen et al., 2025). Genomic coordinates are shown below each track, with promoter regions highlighted in yellow.

Fig.S5. Spatiotemporal correlation analysis of *in vitro* differentiation trajectories mapped to embryonic reference.

A-J: PCC heatmaps comparing *in vitro* differentiation transcriptomes (**A-E**: WT; **F-J**: p-Enh-KO) against *in vivo* E5.5 and E7.5 embryonic coordinates. Each spot represents PCC values between differentiation transcriptomes and embryonic spatial domain.
K-N: Spatiotemporal PDI heatmaps comparing p-Enh-KO and WT samples

across *in vitro* differentiation stages, digitally predicted by ST-Pheno at E7.5 embryonic regions. EA: Anterior endoderm; EP: Posterior endoderm; MA: Anterior mesoderm; MP: Posterior mesoderm/Primitive Streak; A/L1/R1: Anterior epiblast/ectoderm; P/L2/R2: Posterior epiblast/ectoderm; PCC: Pearson correlation coefficients.
