# Supplementary material for: Posterior enhancer (p-Enh) maintains early neuromesodermal progenitors bi-potency during gastrulation
Source: Cell Regen. 2025 Nov 15;14:44. doi: 10.1186/s13619-025-00272-8 (PMC12618760; doi:10.1186/s13619-025-00272-8)
Supplement: Supplementary file 2 — Supplementary Material 2: Table S1. List of quantitative PCR primer sequences. Table S2. List of primary antibodies and their applications. Table S3. Target sequences of sgRNAs for generating p-Enh-KO cell lines. [file 13619_2025_272_MOESM2_ESM.docx]

**Table_S1_primer:**

| Primer name | Primer sequence (5' to 3') |
| --- | --- |
| Tbx6_Forward | ATGTACCATCCACGAGAGTTGT |
| Tbx6_Reverse | GGTAGCGGTAACCCTCTGTC |
| Pax6_Forward | TACCAGTGTCTACCAGCCAAT |
| Pax6_Reverse | TGCACGAGTATGAGGAGGTCT |
| Zfp42_Forward | CCCTCGACAGACTGACCCTAA |
| Zfp42_Reverse | TCGGGGCTAATCTCACTTTCAT |
| Hoxa5_Forward | AAGCAAGAAAGAGCAGCTTGC |
| Hoxa5_Reverse | GTGCCAATGTGTGTGTTTC |
| Gapdh_Forward | AGGTCGGTGTGAACGGATTTG |
| Gapdh_Reverse | TGTAGACCATGTAGTTGAGGTCA |
| T_Forward | GCTTCAAGGAGCTAACTAACGAG |
| T_Reverse | CCAGCAAGAAAGAGTACATGGC |
| Mesp1_Forward | GTCACTCGGTCCTGGTTTAAG |
| Mesp1_Reverse | ACGATGGGTCCCACGATTCT |
| Sox2_Forward | GCGGAGTGGAAACTTTTGTCC |
| Sox2_Reverse | CGGGAAGCGTGTACTTATCCTT |
| Eomes_Forward | GCGCATGTTTCCTTTCTTGAG |
| Eomes_Reverse | GGTCGGCCAGAACCACTTC |
| Mixl1_Forward | ACGCAGTGCTTTCCAAACC |
| Mixl1_Reverse | CCCGCAAGTGGATGTCTGG |
| Klf4_Forward | GTGCCCCGACTAACCGTTG |
| Klf4_Reverse | GTCGTTGAACTCCTCGGTCT |
| Nanog_Forward | TCTTCCTGGTCCCCACAGTTT |
| Nanog_Reverse | GCAAGAATAGTTCTCGGGATGAA |
| Pou5f1_Forward | GGCTTCAGACTTCGCCTCC |
| Pou5f1_Reverse | AACCTGAGGTCCACAGTATGC |
| Cdx2_Forward | CAAGGACGTGAGCATGTATCC |
| Cdx2_Reverse | GTAACCACCGTAGTCCGGGTA |
| Nkx1-2_Forward | CGCGCACTGCCTTCACTTA |
| Nkx1-2_Reverse | GCGTGGCTCGAAACTTGTT |
| Fgf8_Forward | CCGAGGAGGGATCTAAGGAAC |
| Fgf8_Reverse | CTTCCAAAAGTATCGGTCTCCAC |
| Hes7_Forward | CGGGAGCGAGCTGAGAATAG |
| Hes7_Reverse | CACGGCGAACTCCAGTATCT |
| Meox1_Forward | GAAACCCCCACTCAGAAGATAGC |
| Meox1_Reverse | TCGTTGAAGATTCGCTCAGTC |
| Hoxb4_Forward | ATTGGGGTTTACCGTGCTCAC |
| Hoxb4_Reverse | CCTGAAGCGGGGTTCCTTG |
| Hoxb9_Forward | GCACGCCCGAGTACAGTTT |
| Hoxb9_Reverse | GGCAGAGGGGTTGGTTTGA |

**Table_S2_antibody:**

| Antibody | Host Species | Supplier | Catalog Number | Dilution |  |
| --- | --- | --- | --- | --- | --- |
| OCT4 | Mouse | Santa Cruz | sc-5279 | 1:200 |  |
| NANOG | Rabbit | Abcam | ab80892 | 1:200 |  |
| SOX2 | Rabbit | Abcam | ab97959 | 1:1000 |  |
| T | Goat | R&D systems | AF2085 | 1:500 |  |
| HOXB9 | Mouse | Santa Cruz | sc-398500 | 1:50 |  |
| SOX1 | Goat | R&D systems | AF3369 | 1:100 |  |
| TBX6 | Goat | R&D systems | AF4744 | 1:100 |  |
| MEOX1 | Rabbit | Invitrogen | PA527967 | 1:500 |  |
| CDX2 | Rabbit | Abcam | ab76541 | 1:500 |  |

**Table_S3_sgRNA and genotyping:**

| Primer names | Sequence (5'-3') |
| --- | --- |
| p-Enh-F1 | TGAAAATACCTGGGATGTGG |
| p-Enh-R1 | GGAAGGAAGTGAGCAAAGTG |
| p-Enh-F2 | CGCCTGCTTTGGAAGTAAAG |
| p-Enh-R2 | CCGGCTTTGATGTACACCTT |
|  |  |
| single guide RNA | sequence (5' to 3') |
| single guide RNA 1 | ggaccttgacaaatagcgcg |
| single guide RNA 2 | tgagcggaggatcgccggta |
